# Gibberellin-mediated internode elongation in grasses with a focus on bamboo: molecular pathways and regulatory networks

**DOI:** 10.3389/fpls.2025.1665328

**Published:** 2025-09-11

**Authors:** Lei Dai, Xiumin Zhao, Shiying Liu, Krishnamurthi Keerthana, Venkatesan Vijayakanth, Yongqi Zhi, Ming Chen, Feng Que, Muthusamy Ramakrishnan, Zishan Ahmad, Qiang Wei

**Affiliations:** ^1^ State Key Laboratory of Tree Genetics and Breeding, Co-Innovation Center for Sustainable Forestry in Southern China, Bamboo Research Institute, School of Life Sciences, Nanjing Forestry University, Nanjing, China; ^2^ Shaoxing Academy of Agricultural Sciences, Shaoxing, China

**Keywords:** gibberellin, hormone interaction, Poaceae, bamboo growth, epigenetic regulation

## Abstract

Internode elongation in Poaceae plants significantly influences stem development and grain yield. Gibberellin (GA), a key hormone, regulates this elongation and overall development. In cereal members of Poaceae, such as rice and wheat, the application of dwarfing genes involved in GA metabolism or signaling pathways during the Green Revolution led to increased grain yield, underscoring GA’s importance in plant breeding. Although bamboo was not a part of this historical context, optimizing its growth requires an understanding of GA-mediated internode elongation control. This review systematically elucidates the molecular framework of GA-regulated internode elongation in Poaceae, with a specific focus on bamboo. It examines GA’s biosynthetic pathway, metabolic regulation, and signal transduction mechanisms. The review also discusses how GA interacts with other hormone pathways to regulate internode growth and suggests future research directions. Finally, this review provides a reference for a deeper understanding of the molecular mechanisms behind GA-regulated bamboo internode growth and its potential application in bamboo breeding.

## 1 Introduction

Poaceae plants (also called Gramineae or true grasses) play a vital and irreplaceable role across various fields. This family includes essential food crops such as rice (*Oryza sativa*), wheat (*Triticum aestivum*), and maize (*Zea mays*), which are fundamental to global food security (Canton, 2021). Additionally, it encompasses economically significant species like Moso bamboo (*Phyllostachys edulis*) and sugarcane (*Saccharum officinarum*), as well as various forage grasses essential for livestock (Ahmad et al., 2021; Raj et al., 2024). Because they are major food crops (rice, wheat, and maize), provide feed for animals, and contribute biomass for the manufacture of materials and bioenergy, Poaceae species are important to the economy and agriculture. Plant height is a critical agronomic trait that directly impacts crop productivity. An optimal increase in plant height enhances light absorption by leaves and promotes biomass accumulation. However, excessive height, particularly in rice, increases the risk of lodging, which can significantly reduce yield. Therefore, maintaining an appropriate plant height is essential for maximizing productivity (Cai et al., 2017). In Poaceae plants, internode elongation primarily determines plant height and is regulated by a complex interplay of genetic and environmental factors. This process is tightly controlled by plant hormones, including gibberellins (GA), auxins (IAA), abscisic acid (ABA), jasmonic acid (JA), and brassinosteroids (BR), all of which play key roles in internode growth (Wang et al., 2022; Zhao et al., 2022; Huang et al., 2025; Miao et al., 2025). Although bamboo was not part of the Green Revolution, it has attracted considerable attention due to its remarkable growth rate, woody perennial nature, and increasing importance as a sustainable biomass source (Ahmad et al., 2021). Bamboo has distinct biological characteristics that set it apart from annual cereals. These include a long vegetative phase, infrequent flowering, and fast internode elongation up to a meter per day in certain species (Chen et al., 2022). In addition to setting bamboo apart from other grasses, these traits also make it a perfect model for investigating new facets of GA-mediated internode elongation control. Understanding the mechanisms of internode elongation in bamboo can uncover conserved and divergent pathways that not only support biomass-focused breeding in bamboo but also offer strategies to optimize plant height and productivity in other Poaceae crops.

Among various phytohormones, GA is the primary regulator of internode elongation and plant height in Poaceae (Wu et al., 2021). GA directly stimulates cell elongation and regulates cell division by interacting with DELLA proteins (Xiong et al., 2023; Ohama et al., 2025). The Green Revolution’s success in developing semi-dwarf cereal varieties, which exhibit reduced GA sensitivity, highlights the significance of controlled GA regulation in enhancing crop yields and lodging resistance (Robil et al., 2024). A plant growth regulator such as paclobutrazol is used to control excessive GA activity and avoid lodging (Desta and Amare, 2021). By preventing GA biosynthesis, paclobutrazol improves root development, strengthens stems, and decreases internodal growth. By adjusting hormonal balances, its use has been demonstrated to maximize yields and improve crop resistance to abiotic challenges (Desta and Amare, 2021). Given its profound influence on crop architecture, lodging resistance, and adaptability to environmental stress, GA remains the most critical hormone for internode elongation research, making it a key target in modern agricultural biotechnology. Beyond agronomic applications, advances in genomics and systems biology have enabled deeper insights into GA-mediated regulation.

**Figure 1:**
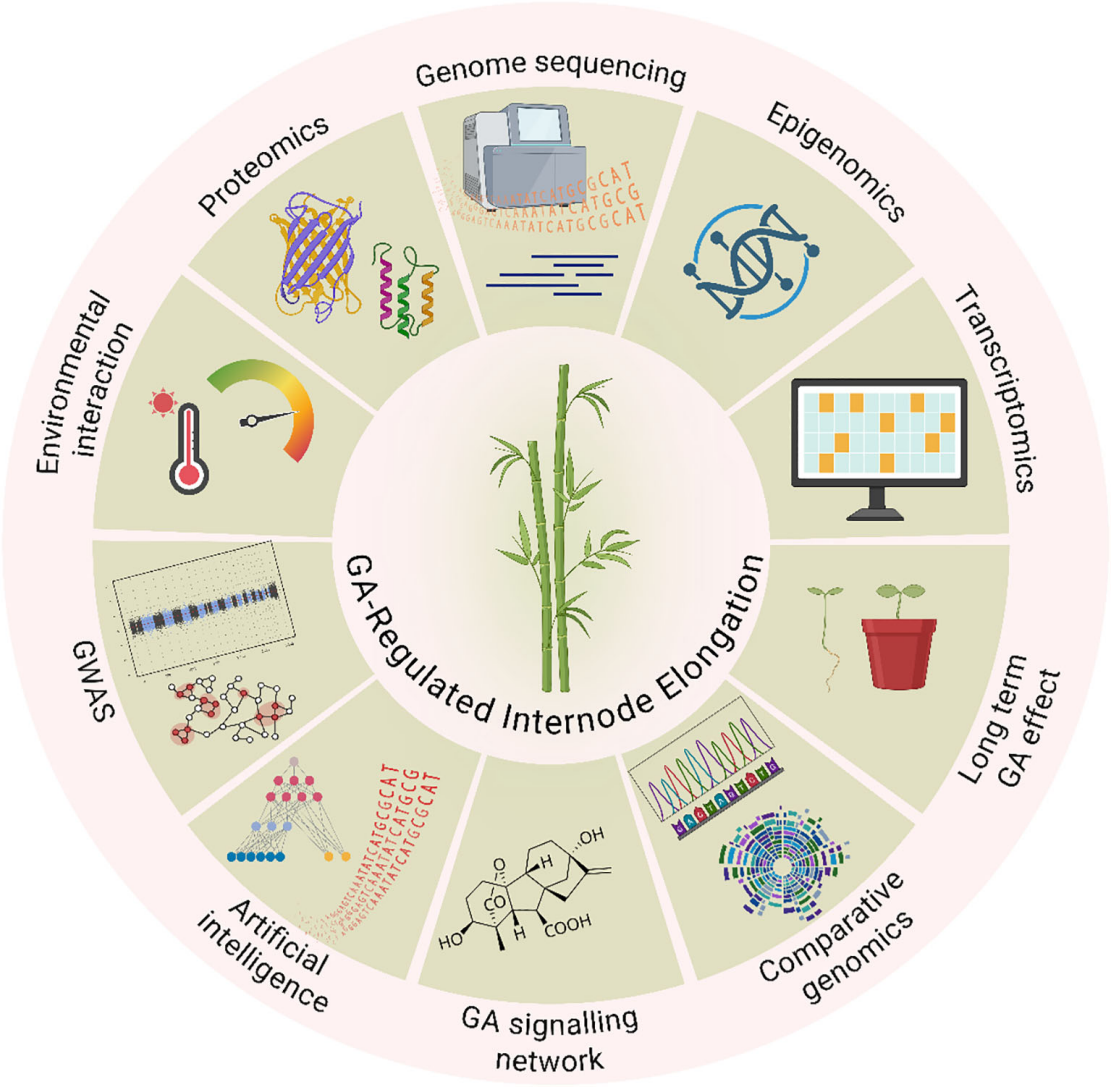
Integrative research framework for understanding gibberellin (GA)-regulated internode elongation in grass species, exemplified by bamboo. The framework illustrates the multi-level approach combining morphological observations, cellular analyses, and molecular profiling to dissect GA-mediated growth regulation. It encompasses GA biosynthesis and signaling pathways, cross-talk with other phytohormones, gene expression networks, and environmental influences affecting internode elongation.

Understanding the function of GAs in plant growth and development has advanced significantly in recent years due to developments of integrative nature of modern plant biology, combining genomics, system biology and computational tools to decode hormone-regulated growth (**Figure 1**). Given the importance of internode growth in determining grass architecture and productivity, a comprehensive understanding of GA biosynthesis, signal transduction, and regulatory mechanisms is essential for improving agronomic traits. Determining the molecular mechanisms governing GA control in bamboo, a perennial grass with rapid growth, presents encouraging opportunities for focused genetic enhancement. It might be feasible to improve structural integrity, maximize biomass yield, and optimize culm height by adjusting GA routes and how they interact with other hormones. These characteristics are critical for both commercial production and ecological sustainability. As a representative model of the Poaceae, bamboo is the subject of this review, which attempts to give a thorough examination of the GA-related mechanisms governing internode elongation with a focus on their possible benefits in sustainable agriculture and bamboo enhancement. Despite extensive knowledge in cereals, the molecular dynamics of GA signaling in perennial grasses like bamboo remain underexplored. Unlike annual cereals, bamboo is a fast-growing perennial grass with significant ecological and economic value, yet the hormonal regulation of its internode elongation remains underexplored. This review synthesizes current knowledge on GA-mediated internode elongation in Poaceae, with a focus on bamboo, and explores how this understanding can inform genetic improvement and sustainable use of bamboo and other grasses. We examine the possible use of GA-related genes in breeding programs, highlight significant hormonal regulation variations between bamboo and conventional grasses, and talk about prospective future research avenues. In addition to increasing the productivity of cereal and forage crops, this integrative method promotes genetic improvement and the sustainable use of bamboo for climate resistance, fiber, and lumber.

## 2 GA biosynthesis and signaling

### 2.1 A brief overview of structure and types of GA

Gibberellin (GA) refers to a class of naturally occurring plant hormones characterized by a tetracyclic diterpenoid structure with carboxylic acid functional groups (Hedden, 2003; Takehara and Ueguchi-Tanaka, 2018). Most members of this hormone family are built upon either the *ent*-gibberellane (C_20_) or the ent-20-norgibberellane (C_19_) carbon skeleton, which form the core structure of bioactive and precursor GA compounds (Hedden, 2003; Urbanova et al., 2012). The structural diversity among gibberellins arises from variations in the number and position of double bonds, hydroxyl groups, and the presence or absence of lactone rings (Hedden and Sponsel, 2015).

According to the different number of C atoms, GA can be divided into C20-GAs and C19-GAs (Rademacher, 2016). C20-GAs contain 20 carbon atoms and act as precursors to bioactive forms (e.g., GA12, GA53). C19-GAs contain 19 carbon atoms, formed by the removal of a C - 20 group from C20-GAs, and include biologically active GAs (e.g., GA_1_, GA_3_, GA_4_, GA_7_) (Takahashi et al., 2018; Hedden, 2020). In addition, GA can be classified into bound and free states based on whether it can be combined with other substances. The bound GA is generally inactive and plays storage and transport functions in plants. Gibberellin can be transformed into each other in the free state and the bound state to meet the requirements of different growth and development stages of plants (Hedden, 2020; He et al., 2020). For example, during seed germination, inactive bound GAs is hydrolyzed into bioactive forms that promote cell elongation and division. At the seed maturity stage, the free GA is continuously transformed into the bound state and stored (Hedden, 2020; Zeng et al., 2024). Moreover, GA can also be divided based on biological activity for example, bioactive GAs directly involved in promoting plant growth by stimulating cell division and elongation (e.g., GA1, GA3, GA4, GA7) and inactive GAs are the form of precursors or deactivated forms of GAs, converted into active forms when required (e.g., GA12, GA20, GA53) (Talon et al., 1990; Hedden, 2020). This structural diversity enables GAs to perform distinct physiological functions, influencing plant height, seed germination, flowering, and stress responses. Understanding the structure and classification of GAs is crucial for elucidating their diverse roles in plant growth and development.

### 2.2 Gibberellin biosynthesis

**Figure 2:**
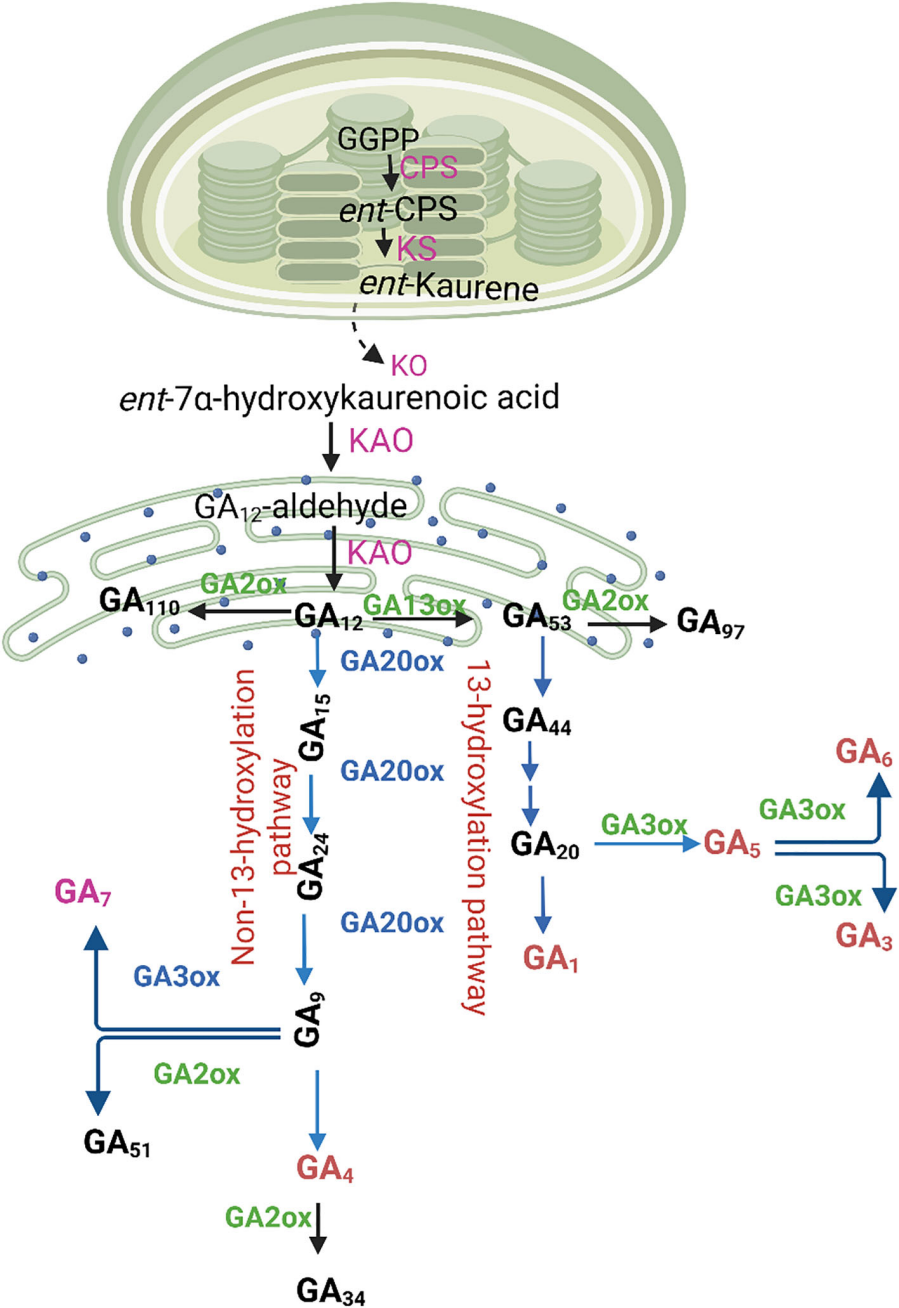
Gibberellin (GA) biosynthesis in higher plants follows a well-organized pathway involving multiple cellular compartments. The process begins in the plastids, where early intermediates are synthesized, leading to the formation of GA_12_ in the endoplasmic reticulum. Once in the cytoplasm, GA_12_ undergoes further modifications catalyzed by GA20-oxidase (GA20ox) and GA3-oxidase (GA3ox), leading to the production of bioactive GAs. These bioactive forms arise through two distinct branches: the non-13-hydroxylation pathway, which produces GA_7_ and GA_4_, and the 13-hydroxylation pathway, yielding GA_5_, GA_6_, GA_3_, and GA_1_. To regulate GA homeostasis, GA2-oxidase (GA2ox) enzymes deactivate both precursors and active gibberellins, preventing excessive accumulation. Key enzymes involved in this pathway include ent-copalyl diphosphate synthase (CPS), ent-kaurene synthase (KS), ent-kaurene oxidase (KO), and ent-kaurenoic acid oxidase (KAO). Their coordinated activity ensures the precise control of GA biosynthesis, allowing plants to regulate growth and developmental processes effectively.

Gibberellins are produced by all vascular plants and several fungal and bacterial species that associate with plants as pathogens or symbionts. Over the past 60 years, research on gibberellin biosynthesis has progressed to provide comprehensive information on the pathways, biosynthetic enzymes, and their genes in all three kingdoms, where hormone production evolved independently, following the initial experiments on the biosynthesis of gibberellic acid in the fungus *Fusarium fujikuroi* (Hedden, 2020). Gibberellin A3, also called gibberellic acid, is the most prevalent gibberellin found in *F. fujikuroi* and the first to be structurally characterized (Curtis and Cross, 1954). Three cell compartments such as stage I: plastid biosynthesis; stage II: ER oxidation; and stage III: cytoplasmic conversion; are thought to be where GA biosynthesis takes place (**Figure 2**).

#### 2.2.1 Stage I: plastid biosynthesis

The process begins with geranylgeranyl diphosphate (GGGP), a common precursor for diterpenoids. In plastids, the initial transition from GGGP to ent-kaurene takes place (Yamaguchi and Kamiya, 2000). In plants, ent-kaurene is formed in plastids, predominantly via the methylerythritol 4-phosphate (MEP) pathway, although there is some contribution from the mevalonic acid (MVA) pathway, presumably dependent on the influx of isoprenoid intermediates of GGPP synthesis into the plastids from the cytosol (Flügge and Gao, 2005). Geranylgeranyl diphosphate (GGPP) is converted to ent-kaurene in the stroma of proplastids or developing chloroplasts, but not in mature chloroplasts. This is probably due to the increased molecular exchange made possible by proplastid inner membranes, which may facilitate terpene pathway crossover (Bräutigam and Weber, 2009). Furthermore, mature chloroplasts, where GGPP is extensively used for the manufacture of carotenoid and chlorophyll, have less competition for GGPP than immature plastids (Zhou et al., 2017).

Seven of the ten functional GGPP synthase (GGPPS) genes in *Arabidopsis* encode enzymes that are in the plastid. In *Arabidopsis*, GGPPS11 is highly expressed but not involved in GA biosynthesis (Beck et al., 2013; Ruiz-Sola et al., 2016). Rather than utilizing the MEP pathway, which primarily supports the synthesis of photosynthetic terpenoids, GA biosynthesis is likely supplied by smaller GGPPS enzymes. These enzymes are thought to be connected with the mevalonate pathway, which may serve as the main source of GGPP for GA synthesis. Rice, in contrast to *Arabidopsis*, has a single functional plastidic GGPPS that is responsible of the production of all plastid diterpenoid compounds, including ent-kaurene (Zhou et al., 2017). The distribution of GGPPS between the stroma (GA biosynthesis) and thylakoid (chlorophyll) is determined by the amount of *OsGRP*. In contrast to *OsGRP*, GA production in *Arabidopsis* and tomatoes requires a functional GPS (Van Schie et al., 2007).

#### 2.2.2 Stage II: ER oxidation

In the second stage, two cytochrome P450 monooxygenases, ent-kaurene oxidase (KO) and ent-kaurenoic acid oxidase (KAO), catalyze the conversion of ent-kaurene to GA12, the first C20-GA on the biosynthesis pathway (Sasaki et al., 2003). In *Arabidopsis*, KO is found on the outer chloroplast membrane and possibly the endoplasmic reticulum (ER), while the two KAOs are specifically localized to the ER. This indicates that as ent-kaurene moves out of the plastid, it undergoes oxidation at the ER, likely facilitated by a membrane interface that enables the transfer of nonpolar metabolites between these organelles (Block and Jouhet, 2015). In rice, there are five KO-like genes, including *OsKO2*, which was demonstrated to have KO action by heterologous expression in yeast (Sakamoto et al., 2004). In *Arabidopsis*, KO is expressed by a single gene. *OsKO2* is implicated in GA-biosynthesis and may be the only gene in the cluster with this function, as mutations in this gene result in severe GA-deficiency and dwarfism. A study utilized CRISPR-Cas9 technology to create knockouts of various KO genes in rice, including *OsKO2* (Chen et al., 2019). The *OsKO2* knockout mutants exhibited severe dwarfism and sterility, indicating a crucial role in GA biosynthesis. The study also found a strong correlation between the expression levels of *OsKO1* and *OsKO2* across different anatomical parts, suggesting that these genes may work together in GA biosynthesis.

#### 2.2.3 Stage III: cytoplasmic conservation

In the third stage, in the cytoplasm, the aldehyde group on the 7th position of GA12-aldehyde is oxidized to form 20-C GA12, which is then catalyzed by GA3 oxidase (GA3ox) and GA20 oxidase (GA20ox) to form GA_1_ and GA_4_ with high biological activity (Hedden and Thomas, 2012; Hedden, 2020). Another study revealed that bio-active GA production may occur in two compartments, the cytosol and the nucleus, which is contradictory to this generally held belief (Chen and Tan, 2015). Interestingly, GA receptor GID1’s (GA-INSENSITIVE DWARF1) localization and this dual-localization occur at the same time (Ueguchi-Tanaka et al., 2005; Willige et al., 2007). Notably, studies have demonstrated that key enzymes involved in GA biosynthesis, such as gibberellin 3-oxidase (GA3ox) and gibberellin 20-oxidase (GA20ox), are dual-localized in both the cytosol and the nucleus. For example; according to a study on maize, both compartments include the D1 protein, which codes for GA3ox (Chen et al., 2014). Both the cytosol and the nucleus may be sites of bioactive GA production and perception, as this dual localization is consistent with the distribution of the GA receptor GID1. These findings imply that the production of bioactive GAs is not confined to a single cellular compartment but may occur in multiple locations within the cell, offering a more nuanced understanding of GA biosynthesis and signaling pathways.

### 2.3 Signaling pathway of gibberellin

The signaling pathway plays an important role in regulating the balance of GAs in Poaceae. Over the last ten years, several variables that are critical for GA sensing have been discovered, mostly through screening for *Arabidopsis* GA-signaling mutants and rice. Specifically, genes encoding the GA receptor GID1 and GID2, DELLA proteins, F-box proteins and AtSLY1 (Hirano et al., 2008; Harberd et al., 2009; Zeng et al., 2024). We have been able to build a model of GA signaling using physiological and biochemical investigations of these proteins (Ueguchi-Tanaka et al., 2005; Wang et al., 2024). This model states that the GID1 receptor interacts to GA when GA is present. The SCFGID2/SLY1 complex then degrades DELLA protein because of interactions between the GID1-GA complex and DELLA proteins, which are negative regulators of GA function. The SCF (SKP1, Cullin, F-box cotaining) complex, which includes the adaptor protein Skp1, the scaffold protein Cullin, an F-box protein such as GID2, and a RING-H2 finger protein (Rbx1), acts as an E3 ubiquitin ligase. This complex facilitates the transfer of ubiquitin molecules to specific target proteins, including the DELLA repressor SLEEPY1 (SLR1), thereby tagging them for proteasomal degradation by the 26S proteasome (Ueguchi-Tanaka et al., 2005). The degradation of DELLA proteins relieves their repression on GA signaling, thus promoting GA-mediated developmental processes such as stem elongation and seed germination.

DELLA proteins are characterized by an N-terminal region containing two conserved motifs, DELLA and TVHYNP—that function as repressors of gibberellin (GA) responses, and a C-terminal GRAS domain, which classifies them as members of the GRAS family of plant-specific transcriptional regulators (Bolle, 2004). Currently, the GA-GID1-DELLA signaling pathway is the most extensively studied and is the most fundamental GA signaling pathway in plants (Ueguchi-Tanaka et al., 2005; Hirano et al., 2008; Shani et al., 2024). DELLA proteins, key regulators linked to Green Revolution traits, modulate plant growth by inhibiting gibberellin signaling and coordinating diverse hormonal and environmental responses (Alabadí and Sun, 2025). They interact with numerous transcription factors to regulate development and stress adaptation. Advances in genetics and structural studies have provided deeper insights into their functional mechanisms and evolutionary significance.

Under low GA concentrations, the DELLA protein SLR1 in rice acts as a repressor of GA signaling, thereby restricting plant growth (Ueguchi-Tanaka et al., 2005). When GA levels increase, the hormone binds to its receptor GID1, forming a GA-GID1 complex that interacts with conserved regions, including the DELLA and TVHYNP domains of SLR1. This interaction stabilizes the GA-GID1-SLR1 complex, which is then recognized by the SCFGID2 E3 ubiquitin ligase complex. GID2, the F-box protein within this complex, facilitates the ubiquitination of SLR1, marking it for degradation via the 26S proteasome. The removal of SLR1 lifts its inhibitory effect, thereby activating downstream GA signaling pathways and promoting plant growth (Nemoto et al., 2017). Different modulators enhance GA responses in addition to this fundamental signaling pathway. Second massengers such as Ca^2+^, cGMP acts as positive regulators, enhancing GA signaling, whereas nitric oxide (NO) negatively regulates the pathway (Acharya, 2024; Li et al., 2024b). However, their functional roles in GA-mediated internode elongation remain largely unexplored in Poaceae. Furthermore, in *Arabidopsis*, MEDIATOR15 (MED15), a component of the mediator complex, has been shown to interact with DELLA proteins, promoting their degradation and GA biosynthesis (Ohama et al., 2025). Similar to DELLA mutants, *MED15* overexpresses, and mutants display GA-related growth abnormalities. By accelerating DELLA turnover and inducing GA biosynthesis, MED15 facilitates DELLA degradation and advances GA signaling. For plants to develop in response to environmental cues like warmth and darkness, this regulation is essential. These results provide possible uses for crop improvement by highlighting the function of MED15 in regulating DELLA stability.

**Figure 3:**
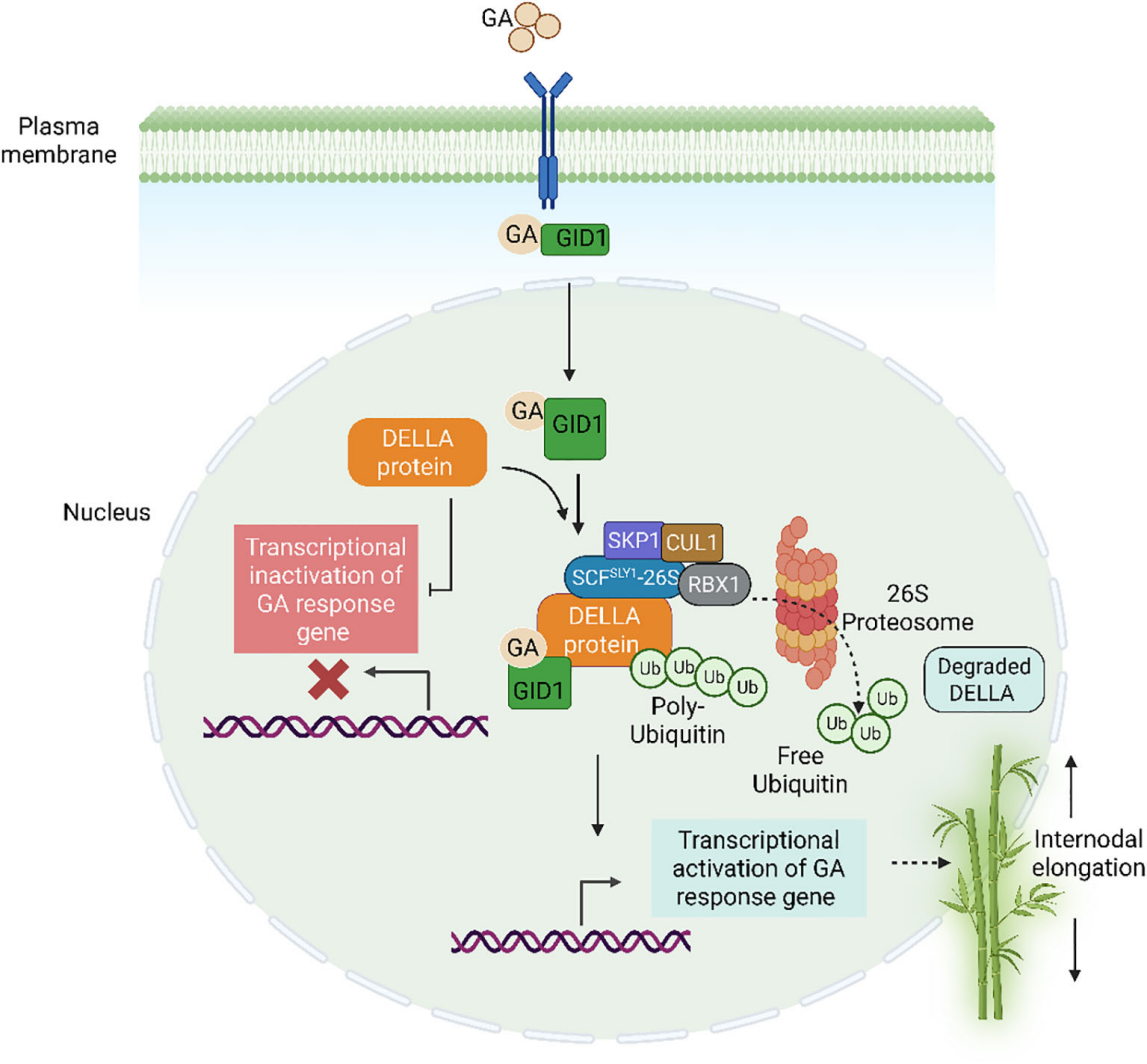
Gibberellin (GA) signaling regulates plant growth by promoting the degradation of DELLA proteins, which act as repressors of GA responses. In the absence of GA, DELLA proteins inhibit transcription factors like PIFs, restricting growth. When GA is present, it binds to the GID1 receptor, forming a GA-GID1-DELLA complex. This complex is recognized by the SCF^GID2/SLY1^ ubiquitin ligase, leading to DELLA polyubiquitination and subsequent degradation via the 26S proteasome. As a result, GA-responsive genes are activated, promoting stem elongation, seed germination, and flowering. Additionally, DELLA proteins integrate crosstalk with other hormones and environmental cues, adding complexity to their regulatory role.

Concurrently, it has been demonstrated that nitrate functions as a signaling molecule and a nutrient that affects plant growth by modifying DELLA protein levels through elevated GA biosynthesis (Camut et al., 2021). DELLA degradation results from nitrate signaling’s enhancement of the expression of GA biosynthesis genes (such as *GA20ox* and *GA3ox*). In turn, this promotes both cell proliferation in a DELLA-dependent manner and cell elongation in a largely DELLA-independent manner by activating GA-responsive genes involved in cell cycle progression (e.g., *CYCLIN D* family), expansion (e.g., *EXPANSINs*), and elongation (Camut et al., 2021; Alabadí and Sun, 2025). The transcriptional activation of growth-promoting genes is thus unlocked by the breakdown of DELLA proteins, which are central repressors of GA signaling. **Figure 3** depicts the DELLA mediated GA signaling.

Studies in wheat (*Triticum aestivum*) have demonstrated that the reduced height (*Rht*)-*B1b* and *Rht-D1b* alleles, which were crucial to the Green Revolution, encode modified DELLA proteins that limit GA responsiveness, leading to shorter stems, better lodging resistance, and more grains (Wheat, 2011). This supports the conserved role of DELLA proteins in GA signaling. The production of DELLA proteins that are unable to bind the GA receptor *GID1* due to additional mutations like *Rht-B1c* (caused by a 30-amino acid insertion in the DELLA domain) and *Rht-D1c* (induced by an increased gene copy number) results in severe dwarfism by preventing breakdown and strengthening growth suppression.

Emerging transcriptome analyses indicate that GA biosynthesis and signaling genes are differentially expressed throughout important developmental stages including shoot elongation and internode expansion in bamboo, despite the fact that genomic and molecular studies are more scarce than in rice and wheat (Chen et al., 2022; Gao et al., 2022). Dynamic regulation of GA-related genes during rapid internode development has been found in transcriptome investigations of bamboo (*P. edulis*), which has one of the fastest known shoot elongation rates among terrestrial plants. A well-coordinated GA signaling network is indicated by the different temporal and spatial expression patterns of genes such *GA20ox*, *GA3ox*, *GID1*, and DELLA homologs (Bai et al., 2023). Interestingly, DELLA expression declines with increasing elongation, indicating increased sensitivity to GA in tissues that are actively developing. These findings are consistent with a conserved but refined GA signaling pathway in bamboo that has been tailored to sustain its distinct growth pattern.

Early in the history of land plants, DELLA proteins underwent gene duplication and neofunctionalization, leading to their diversification, particularly in angiosperms (Briones-Moreno et al., 2017; Phokas and Coates, 2021). Their spread throughout the Poaceae family, which includes bamboo and wheat, allows for precise GA regulation in a variety of tissues and settings. Stress adaptability and phenotypic plasticity are supported by this functional diversification. Knowing the evolution of DELLA provides useful targets for agricultural improvement in climate change.

Numerous mechanisms, including transcriptional regulation through MED15, second messengers including Ca^2+^, cGMP, and NO, and the GA–GID1–SCF-mediated ubiquitin-proteasome system, intimately regulate this process (Mao et al., 2025a; Ohama et al., 2025). Furthermore, nitrate signaling adjusts plant design and resource allocation by combining hormonal pathways and nitrogen availability. A strategic framework for crop development is provided by an understanding of these regulatory processes. Optimized growth responses, enhanced stress resilience, and adaptive architecture that can adjust to changing environmental conditions can all be achieved by targeting DELLA stability using hormonal or environmental signals.

## 3 Internode growth in bamboo: how it is distinct from conventional grass models?

**Figure 4:**
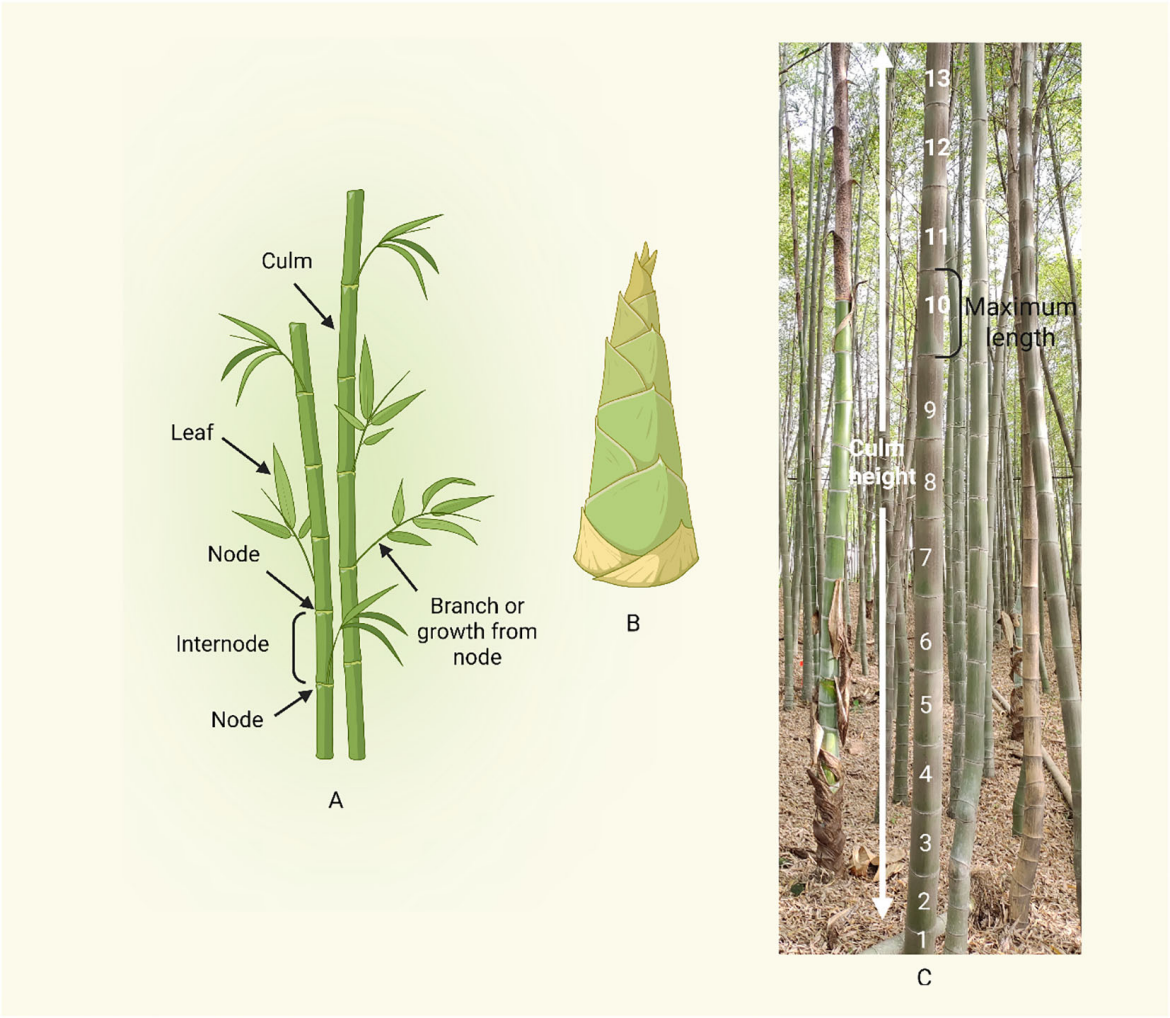
Bamboo culm morphology and growth pattern. **(A)** Basic morphology of a bamboo culm, focusing on the alternating pattern of nodes and internodes. **(B)** Bamboo shoot (Young culm bud). **(C)** Culm height and internode elongation in bamboo. Internodes 1 – 8 represent the actively elongating region, contributing to rapid vertical growth. Internode 9 – 10 marks the maximum elongation regio2.2.2n, after this point, internode elongation slows, the “maximum length” label at internode 10 suggests a transition zone between rapid elongation and growth cessation.

Bamboo’s distinct structural and regulatory characteristics set it apart from other types of grass in terms of internode growth. Despite being a member of the Poaceae family and having monocot traits in common with other grasses, bamboo differs from other grasses in terms of physiological complexity and developmental dynamics due to its growth strategy, especially the quick elongation of culms (**Table 1**; **Figure 4**). Bamboo has a burst-type growth pattern, frequently reaching several centimeters of elongation every day during the peak phase, in contrast to many grasses, which have comparatively stable and modular development (Wang et al., 2025). Cell elongation in the elongation zone, which is more geographically and temporally defined in bamboo than in normal grasses, and cell division in the intercalary meristem interact in a well-coordinated manner to promote this rapid internode elongation (Gao et al., 2022; Guo et al., 2024). Bamboo internodes are anatomically strong and flexible during rapid vertical extension because of their highly lignified outer area and interior parenchymatous tissue.

**Table 1:** Comparative overview of internode growth characteristics in bamboo vs. conventional grass species.

Characteristics	Bamboo	Conventional grasses	References
Growth rate	Rapid elongation (up to 1 m/day	Moderate and steady	(Chen et al., 2022)
Growth pattern	Burst-type/slow-fast-slow	Continuous, modular	(Chen et al., 2022; Wang et al., 2025)
Elongation zone	Well-defined, large elongation zone	Small and less distinct	(Wei et al., 2019; Gao et al., 2022)
Cellular Activities	Strong intercalary meristem activity followed by elongation and lignification	Shorter duration and extent of elongation	(Wei et al., 2019; Guo et al., 2024)
Hormonal regulation	GA, auxin and BR show synergistic gradient along internode	GA- dominant, less pronounced interaction with auxin or BR	(Wang et al., 2018; Bai et al., 2023)
Nutrient source	Heavy reliance on remobilized reserve from rhizome	Mainly dependent on current photosynthesis	(Wang et al., 2025)
Vascular structure	Thick-walled fibers; dense vascular bundles	Fewer vascular bundles; less mechanical specialization	(Tsuyama et al., 2023; Li et al., 2024a)
Gene expression profile	High expression of *WRKY*, *Bzip*, *NAC*, *expansins*, *XTHs*	Less pronounced transcriptional complexity during elongation	(Chen et al., 2021; Lan et al., 2021)
Environmental Response	Strong seasonal influence on phase	More responsive to photoperiod and flowering cues	(Dutta et al., 2018)
Mechanical strength	High due to lignification during elongation	Generally, develops after growth phase	(Adier et al., 2023; Xu et al., 2024)

Moso bamboo (*Phyllostachys edulis*), one of the most widely distributed and economically significant bamboo species in China, is renowned for its exceptionally rapid culm elongation. Its height growth follows a distinct “slow-fast-slow” pattern, where internode elongation progresses sequentially from the base upwards in a similar phased manner (Chen et al., 2022). One of the plants that grows the fastest on the planet is bamboo. In a single day, Moso bamboo can grow about a meter (Chen et al., 2022). This rapid growth is primarily driven by a combination of unique cellular, hormonal, and structural adaptations (Peng et al., 2013; Chen et al., 2022). In the development process of bamboo, cell division dominates in the early stage of bamboo stem development, while cell elongation dominates in the later stage of development. The basal part of bamboo internodes develops and matures first, gradually maturing upwards. The number of nodes is nearly certain prior to the shoots emerging from the soil. Following that, cell division and elongation of the intermediate meristematic issue cause internodes to extend (Wang et al., 2021).

The foundation for culm internode elongation is the fast extension of the parenchyma cells found inside and between vascular bundles of bamboo internodes (Wei et al., 2019). The bamboo culm’s height first increases gradually. After that, bamboo roots start to develop into a root system and the internodes’ growth beneath the soil stops. The culm internodes’ growth rate increases concurrently, peaking at that point. At the end, the young culm’s growth slows down until it reaches the end, its top bends, and its branches spread quickly. As internodes lengthen, young culms are created. How do the internodes of bamboo culms elongate? Internode elongation rates varied with both height and culm position. As bamboo matured, a larger proportion of internodes contributed to overall culm height, offering new insights into the mathematics of bamboo growth (Tan et al., 2024). A study established the growth model of the third internode of the tufted bamboo *Bambusa multiplex* and found that the internode elongation of this species conforms to the logistic model and spiral growth pattern. Similar growth patterns were reported in *Phyllostachys edulis* and *Pseudosasa japonica* suggesting that spiral growth is common in bamboo shoot development (Wei et al., 2019). The two parts at the bottom of the *Bambusa multiplex* internodes are the 1cm cell division zone located at the bottom, and the 1cm cell elongation zone. The internode above the elongation zone is the mature zone, where the cells have already grown (Wei et al., 2019).

Although gibberellins play a key role in encouraging elongation in all grasses, bamboo has a more intricate control. According to studies, the elongation zone exhibits high expression of GA biosynthetic genes (*GA20ox*, *GA3ox*) and GA signaling components (*GID1, SLR1*), frequently in combination with auxin-responsive genes and brassinosteroid signaling genes like BRASSINAZOLE-RESISTANT 1 (*BZR1*) and DWARF4 (*DWF4*) (Wang et al., 2018; Bai et al., 2023). This suggests that bamboo and traditional grasses have higher synergistic hormonal interaction. Furthermore, bamboo uses a mechanism not as commonly used by annual grasses, the remobilization of nutrients and carbohydrates from mature culms and underground rhizomes to drive rapid internode elongation (Zheng et al., 2022; Yuan et al., 2024). With closely spaced vascular bundles and thick-walled fibers, the vascular architecture of bamboo internodes promotes both mechanical stability and effective long-distance transfer of hormones and photo assimilates. There are also differences in environmental responsiveness. Although temperature and photoperiod affect both bamboo and grasses, bamboo closely synchronizes internode elongation and shoot emergence with seasonal cues instead of reproductive time (Lin et al., 2022; Chan et al., 2024). This enables a coordinated growth spurt that is optimized for structural investment and resource allocation. In conclusion, the rate and coordination of development, complex hormonal regulation, vascular adaptations, and gene expression networks of bamboo’s internode growth strategy differ significantly from those of traditional grass models. Knowing these distinctions provides important information about the evolutionary adaptations of perennial monocots and opens the door to using the growth principles of bamboo to increase the biomass and resilience of cereal crops.

## 4 Physiological effects of GA regulation on internode growth in Poaceae plants

Gibberellins (GAs) play a crucial role in regulating internode growth in Poaceae plants by promoting cell division and elongation, thereby influencing plant height, architecture, and overall biomass accumulation (Robil et al., 2024) (**Table 2**). The degree of stem elongation is determined by the interplay between GA signaling and DELLA proteins, with GA-mediated DELLA degradation promoting quick internode growth. Because optimal internode elongation improves light uptake, nutrient delivery, and stress adaptation in in rice, wheat, and maize, this regulation is essential for increasing crop yield (Feiz et al., 2024). For example; exogenous GA_3_ treatment on Moso bamboo seedlings showed that GA_3_ could lead to an increase in plant height, internode elongation, and lignin accumulation (Zhang et al., 2018). A recent study employs spatial and single-nucleus transcriptomics to unravel gene regulatory networks driving rapid bamboo shoot growth, focusing on procambium differentiation, intercalary meristem formation, and vascular development (Guo et al., 2024). Key findings suggest intercalary meristems originate from surrounding parenchyma cells, with specific gene expression patterns linked to hormone signaling and lipid metabolism. Furthermore, procambium and associated meristem cells preferentially express three homologs of *GA2ox* implicated in gibberellin production (*GA2ox1*: *PH02Gene08244*, *GA2ox4*: *PH02Gene48911*, and *GA2ox6: PH02Gene48911*).

**Table 2:** GA-regulated genes involved in internode or shoot elongation across bamboo, rice, wheat, and other Poaceae species.

Gene type	Bamboo	Rice	Wheat	Other Poacea plant such as maize, sorghum etc.	References
GA biosynthesis genes	*PheGA20ox, PheGA3ox* (highly expressed in elongating internodes)	*OsGA20ox1, OsGA3ox2* (critical for elongation)	*TaGA20ox1, TaGA3ox2*	*ZmGA20ox, SbGA20ox*	(Ayano et al., 2014; Izydorczyk et al., 2018; Zhang et al., 2018; Paciorek et al., 2022; Bai et al., 2023; Wang et al., 2023)
GA inactivation genes	Not well studied; possible orthologs exist	*OsGA2ox; OsABF1* (negative regulator)	*TaGA2ox*	*ZmGA2ox, SbGA2ox*	(Shimada et al., 2006; Huang et al., 2010; Cantoro et al., 2013; Tang et al., 2021; Tian et al., 2022; Deng et al., 2024)
GA receptor genes (signaling)	*PheGID1*, expressed in elongation zones	*OsGID1*	*TaGID1*	*ZmGID1, SbGID1*	(Ueguchi-Tanaka et al., 2008; Rodríguez et al., 2012; Li et al., 2013; Ren et al., 2021)
GA-auxin crosstalk	*PheARF47- PheGA20ox3/6* (direct regulation)	*OsARF1/19* interacting with GA pathway	*TaARF2/3* interacting with DELLA	NA	(Chen et al., 2022; Bai et al., 2023; Lee et al., 2023; Sokolowska et al., 2025)
GA-BR integration	*BZR1, DWF4* (co-expressed with GA genes in elongation zone)	*OsBZR1, OsDWF4*	*TaBZR1, TaDWF4*	*ZmBZR1, ZmDWF4*	(Tong et al., 2014; Daviere and Achard, 2016; Li et al., 2018; Zhang et al., 2018; Huang et al., 2022; Milner et al., 2022)
Transcription factors	*PheWRKY*, *bZIP*, *NAC* families (internode-specific), *GATA*	*ERF11*	*TaNAC018-7D, GAMyb*	*KNOTTED1, ZmGRF*	(Chen et al., 2001; Xie et al., 2006; Bolduc and Hake, 2009; Peng et al., 2013; Xu et al., 2015; Wang et al., 2019; Chen et al., 2022; Yu et al., 2023; Chen et al., 2025)
*DELLA* repressors	*PheSLR1* (homolog of DELLA protein)	*OsSLR1*	*Rht-B1/Rht-D1* (dwarfing alleles)	NA	(Ueguchi-Tanaka et al., 2008; Li et al., 2013, 2013b; Shou et al., 2020)

Numerous studies have proved that the dynamic changes in gibberellin levels are closely related to the rapid growth of plants (Peng et al., 2024). The rapid growth of Moso bamboo was examined and internode 18 was found to be a crucial location of high cellular activity (Chen et al., 2022). It produces millions of cells and large levels of lignin and cellulose every day, with discrete zones for cell division, elongation, and secondary cell wall thickening. Growth is significantly influenced by hormonal control involving gibberellin, auxin, cytokinin, and ABA. Additional factors that contribute to bamboo’s remarkable elongation include mechanical pressure and gene control. At present, research has confirmed that the elongation of internodes in Poaceae plants is controlled by two regulatory mechanisms: one is by enhancing cell division in the meristematic region; the second is to achieve cell elongation by adjusting the direction of microtubules, assembling cell wall polymers, and synthesizing and transporting new cell walls (Todaka et al., 2012). In addition, a study dealing about how stem elongation starts in Moso bamboo and found that GAs, auxins, and cytokinins mostly build up in the shoot apex, which is where active growth starts (Gamuyao et al., 2017). Rapid growth start was supported by the apical region’s strong expression of genes linked to DNA synthesis, cell creation, and meristem maintenance. The basal stem, on the other hand, had lignification-related genes and stress hormones. These results imply that early bamboo stem elongation is primarily triggered by GA-enriched apical zones.

Optimizing plant height, biomass, and yield in Poaceae species requires an understanding of the hormonal and molecular mechanisms controlling internode elongation, especially the function of gibberellins and their interaction with DELLA proteins. Moso bamboo’s distinct cellular activity and hormone-driven elongation zones make it a useful model for studying rapid internode growth. The coordination of rapid culm growth via hormone signaling and structural gene regulation is further elucidated by recent transcriptomic and physiological investigations.

### 4.1 GA and cell division in the meristem

GAs control many facets of growth and development, most notably cell division in meristematic tissues (Achard et al., 2009; Tang et al., 2025). GA signaling has been demonstrated to increase cell proliferation rates in the shoot apical meristem (SAM), which in turn promotes overall plant growth. GAs also affect root architecture and adaptation in the root apical meristem (RAM) via regulating cell division activity. A recent and advanced study, that developed a radiometric GA signaling biosensor by engineering a modified DELLA protein that enables real-time detection of GA activity without interfering with transcriptional regulation (Shi et al., 2024). The discovery helped to monitor the GA distribution across different plant tissues. For instance, significant GA activity was seen in the boundary cells separating organ primordia that subsequently develop into internodes. Differential GA signaling patterns have also been found in the growing floral organs, expanding leaves, and root elongation zones. These findings provide important information about how growth and development are regulated at the tissue level.

Functional analysis revealed that GA regulates cell division plane orientation, contributing to internode specification in the SAM. These findings highlight GA’s crucial role in shaping shoot architecture by coordinating cell division and internode formation. In intercalary meristem, GA has been found to be the main hormone that promotes the division of cells which are characteristics of certain monocots like Poaceae species (Wang et al., 2022). In an initial study, it was observed that immersing the internode of deep-water rice in a GA solution or water shortened the cell division cycle of the IM in the uppermost internode from 24 hours to 7 hours. This led to a two-fold increase in the number of cell divisions compared to the control (Bleecker et al., 1986). In another study, it was observed that gibberellin treatment influenced the transition from the G1 to S phase during interphase by inducing the expression of period-specific genes *OsCDC2–2* and *Histone H3*. Additionally, during the G2 phase, GA treatment increased the transcription levels of two key cell cycle genes, *OsCYC1* and *OsCYC2* (Lorbiecke and Sauter, 1998). These findings suggest that GA promotes cell division by shortening the duration between the G1 and S phases.

In another monocot species, node count and internode length, which are controlled by intercalary meristem development and cell elongation, govern maize height. A dwarf mutant called *zm66* was found while screening EMS-induced maize mutants (Wang et al., 2022). This mutant is caused by a mutation in *TERMINAL EAR 1* (*ZmTE1*), which results in shorter internodes and more nodes because it disrupts cell division and elongation. Auxin signaling and cell cycle-related genes were shown to be dysregulated by transcriptome analysis. ZmWEE1 (Zea mays Wee1 kinase) limits ZmTE1 activity to the nucleus, which inhibits cell division, and ZmPP2Ac*-*2 phosphatase encourages dephosphorylation and cytoplasmic localization, which increases cell division. These results demonstrate that ZmTE1 is an essential modulator of plant height and internode growth in maize (Wang et al., 2022). The findings demonstrated the possibility of involvement of GA, because it is known to stimulate intercalary meristem activity; controls auxin signaling and cell cycle- related genes and modulate kinase and phosphatase activities.

GA levels are typically low in SAM cells, maintaining meristem identity, while high GA promotes lateral organ growth (Hay et al., 2002; Shani et al., 2006). However, recent studies show that GA also increases SAM size during floral transition by stimulating cell division and expansion in inner SAM tissues (Hay et al., 2002). DELLAs restrict meristem size by regulating the cell-cycle inhibitor KRP2 in the rib zone (Serrano-Mislata et al., 2017). GA biosynthetic and catabolic genes are differentially expressed in lateral organs, indicating complex spatial GA regulation. These findings highlight GA’s dual role in maintaining SAM identity and promoting meristem expansion. A novel GA signaling biosensor was developed using a mutated, yet GA-sensitive RGA fused to a fluorescent protein, enabling precise quantification of GA signaling activity (Shi et al., 2024). This biosensor revealed how GA regulates cell division orientation in the SAM epidermis, influencing internode formation. This information is very useful for crop improvement plans since it may be possible to create crops with optimal plant height, better biomass allocation, and increased environmental adaptability by modifying GA signaling components or their downstream targets. Breeding efforts aimed at ideotype creation and yield optimization in Poaceae species should benefit greatly from such strategies.

### 4.2 GA and cell elongation

By modifying several cellular processes, GAs plays a critical role in encouraging cell elongation in plants. One main mechanism is to induce expansin (EXP), which are protein that loosen the cell wall to facilitate elongation. Gas also enhance the activity of xyloglucan endotransglycosylases (XETs), enzymes involved in restructuring the xyloglucan-cellulose network in the primary cell wall. In addition, GAs promote the vertical alignment of microtubules along the longitudinal axis of cells, facilitating directional growth (Kushwah et al., 2020; Qiao et al., 2022). Expansins disrupt hydrogen bonds between cellulose microfibrils and xyloglucans, facilitating cell wall relaxation and extension. Concurrently, XETs remodel xyloglucans by cleaving and rejoining them, allowing cellulose microfibrils to separate without compromising cell wall integrity (Sivan et al., 2024). Expansin proteins mainly exist in the internode intercalary meristem and elongation zone and can break the hydrogen bonds between cell wall polymers, such as the cellulose-hemicellulose network that provides the basic mechanical structure for the cell wall, causing the cell wall to relax and ultimately leading to cell elongation (Cho and Kende, 1997; Cosgrove, 2024). For example, following GA treatment and flooding, the mRNA level of *OSEXP4* rises, and this is followed by an increase in internode development (Majda and Robert, 2018). In addition, five β-expansin genes were expressed in the elongation zone of rice internodes, and their expression was induced by GA_3_ treatment (Lee and Kende, 2001).

Currently, 58 EXP genes have been identified in the rice genome database, and 43 EXP genes have been identified in bamboo (Ma et al., 2021). In wheat, there are as many as 241 EXP genes. Gibberellin regulates the expression and activity of cell wall structure modifying enzymes, thereby regulating cell wall elongation (Sauret-Güeto et al., 2012). XET protein plays a key role in the synthesis of cell wall component xyloglucan. It can integrate newly synthesized xyloglucan fragments into the existing xyloglucan network in the cell wall (Nishitani and Tominaga, 1992; Vissenberg et al., 2000), making the structure of the cell wall more loose, thus increasing the extensibility of the cell wall and creating conditions for cell elongation. In a recent study; using the GA biosensor *GPS1*, a GA gradient was observed in dark-grown but not light-grown hypocotyls (Griffiths et al., 2024). *COP1* and *HY5* were identified as key regulators of GA distribution, with *GA20ox1* expression controlling the GA gradient in darkness. The improved *GPS2* biosensor revealed a new pattern of GA depletion during light exposure, explaining hypocotyl growth resetting. *GPS2* offers a powerful tool for studying GA dynamics across various plant conditions and species.

**Figure 5:**
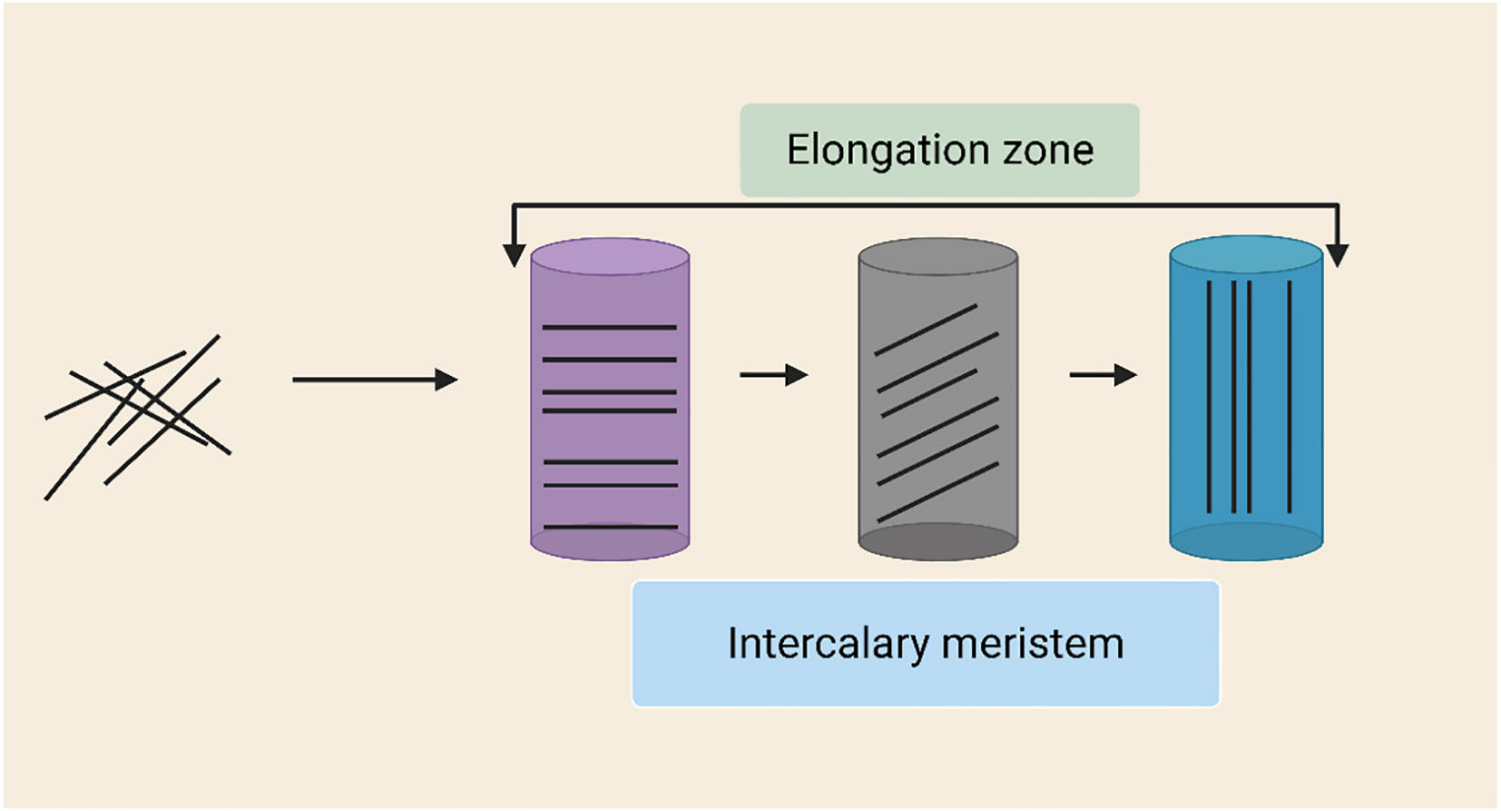
Dynamic microtubule (MT) orientation in the intercalary meristem. As elongation increases, MTs in the intercalary meristem change from random to oblique or longitudinal orientations. In order to guarantee mechanical stability and appropriate vascular differentiation, this reorganization controls cell proliferation, anisotropic growth, and cellulose microfibril deposition.

One important factor in cell elongation in elongating grass internodes is the transverse orientation of cortical microtubules (MTs) mediated by GA (Nemoto et al., 2004). The developmental chronology of cortical MT organization in maize internodes was examined using fluorescence microscopy. Internodal cells had reorientation to a transverse pattern, which remained stable, whereas undifferentiated cells initially had randomly organized MTs. Nodal cells continued to be arranged randomly, but the intercalary meristem kept this transverse MT orientation. Cell fate establishment may be indicated by this MT reorientation during internode initiation (**Figure 5**). Together, these findings underscore the complexity of GA-mediated cell elongation, integrating hormone signaling, cell wall dynamics, and cytoskeletal organization. The coordinated regulation of expansins, XETs, and MT orientation highlights the finely tuned mechanisms that drive internode elongation in grasses. Future research into the molecular regulation of these processes will provide deeper insights into plant growth and adaptability under varying environmental conditions.

## 5 Molecular mechanism of GA regulating internode growth in Poaceae plants

### 5.1 GA synthesis and metabolic related gene regulation of internode growth

Endogenous GA concentration and the sensitivity of GA receptors tightly regulate internode elongation in Poaceae plants; alterations in either factor can significantly impact plant height. By encouraging cell division and growth, gibberellins are essential for promoting stem elongation. Endogenous GA levels can be dramatically lowered by disturbances in GA production or metabolic pathways, which can lead to dwarf phenotypes and stunted growth, potentially affecting crop productivity (Sasaki et al., 2002). Advances in genomic sequencing have made it easier to identify genes associated to GA, especially in model species like *Arabidopsis*. This enabled the identification of key biosynthetic and signaling genes. Understanding the molecular mechanisms governing GA regulation in internode growth is crucial for improving crop yield and biomass accumulation. The primary aim is to dissect the genetic and biochemical pathways underlying GA-regulated internode elongation, with the objective of identifying key regulatory genes and their interactions in different Poaceae species.

During the “Green Revolution”, the discovery and application of the semi-dwarfing variety *SD1* (*Semi-Dwarfing 1*) greatly increased the yield of rice and wheat all over the world. The semi-dwarfing trait is controlled by the GA biosynthesis gene *SD1* in rice and the signaling gene *Rht* in wheat (Sasaki et al., 2002). The gene *SD1* in rice encodes the *GA20ox2* involved in GA synthesis, and mutations in this gene lead to plant dwarfing (Dong et al., 2024). In addition, most mutants or knockouts of GA biosynthesis genes, including *CPS*, *KS*, *KAO*, *KO*, *GA3oxs*, also exhibit dwarfing, which leads to improved lodging resistance (Sakamoto et al., 2004; Qin et al., 2013). Further research showed that the wild-type phenotype could be restored by exogenous application of GA (Sakamoto et al., 2004). GA metabolism also regulates internode elongation. In rice, overexpression of *GA2ox6* leads to dwarfism by accelerating the catabolic inactivation of bioactive GAs and their precursor in the internodes, leading to the limited GA availability for cell elongation and internode expansion (Huang et al., 2010). When the GA catabolism gene *OsGA2ox1* is expressed under the control of the constitutive actin promoter, the resulting plants are dwarfed and grain yield is reduced, as GA is essential for flower development (Sakamoto et al., 2003).

The genes *Rht-B1* and *Rht-D1* genes in wheat encode mutant DELLA proteins that are resistant to GA-induced degradation by the GA receptor GID1, thereby repressing gibberellin signaling and resulting in reduced stem elongation and dwarf phenotypes (Peng et al., 1999). DELLA proteins act as inhibitors of GA signaling and are disrupted by GA, conferring dwarfing traits to mutant wheat. In maize, genes for GA biosynthesis and signaling defects include *anther ear1*, *dwarf1* (*d1*), *d3*, *dwarf8* (*d8*) and *d9* showed varying degrees of plant height reduction (Winkler and Helentjaris, 1995). Maize has six COI proteins (COI1a, COI1b, COI1c, COI1d, COI2a, COI2b) involved in jasmonic acid signaling (Feiz et al., 2024). Mutant analysis revealed that *coi2a coi2b* causes pollen inviability, while *coi1-4x* leads to shorter internodes, reduced photosynthesis, and GA repression via DELLA protein accumulation. *COI* proteins connect jasmonic acid to GA signaling by promoting proteasome-dependent DELLA degradation. Numerous GA-inducible genes that are downregulated in *coi1-4x* encode photosynthetic enzymes that are specific to C4 and are abundant in mesophyll and bundle sheath cells. The buildup of DELLA, which inhibits the expression of genes responsive to GA, including those involved in photosynthesis, is probably the indirect cause of this repression. It is yet unknown, nevertheless, if DELLAs control these C4 genes directly or via secondary transcriptional pathways. Functional diversity among maize *COI* proteins in the regulation of growth and defense is suggested by localization and interaction assays.

Using genomic, morphological, physiological, and transcriptomic data from 11 bamboo species with different ploidies, a study investigated the genetic and evolutionary underpinnings of woody bamboos’ rapid shoot growth. Particularly in *Dendrocalamus sinicus*, gibberellin A1 and two important GA-related genes, *KAO* and *SLRL1*, were found to be important regulators of shoot elongation and culm growth (Mao et al., 2025b). Subgenome asymmetry was demonstrated by the expression of these genes, with subgenomes A and C predominating in giant bamboos and B and D dominating in smaller species. Subgenome-specific characteristics like UTRs and promoter architecture were connected to this imbalance. The results offer fresh perspectives on the roles that gene control and all polyploidization play in woody bamboos’ quick growth.

Apart from the well-established genes implicated in GA manufacture and signaling, like the *Rht-B1* and *Rht-D1* loci that encode DELLA, current research has discovered new regulatory elements that further complicate the regulation of internode elongation in Poaceae. A study introduces two novel antagonistic regulators of internode elongation for example; *ACCELERATOR OF INTERNODE ELONGATION 1* (*ACE1*) and *DECELERATOR OF INTERNODE ELONGATION 1* (*DEC1*), adding a new layer of regulation beyond GA biosynthesis and signal transduction (Nagai et al., 2020). GA and these two antagonistic factors found to control stem elongation in rice. GA-induced internode elongation is made possible by *ACE1*, which stimulates cell division in the intercalary meristem, whereas *DEC1*, a zinc-finger transcription factor, inhibits elongation when it is increased. In both cultivated and wild rice, this regulatory mechanism which is retained throughout the Poaceae family has impacted plant height selection, with taller plants adapting to deep-water conditions and shorter plants developed for lodging tolerance. While *ACE1* and *DEC1* have been functionally characterized in rice, it is still unclear whether their orthologs exist in other members of the Poaceae family, like bamboo, and what their conservation and functional functions are throughout the family. Beyond biosynthesis and signal transduction, these results broaden our knowledge of GA-regulated internode development. Moreover, it connects historical plant height selection in rice domestication and wild adaptation to these regulators, highlighting their evolutionary and agronomic significance (Nagai et al., 2020). In order to optimize plant height under various environmental conditions, breeding programs or genetic engineering can benefit from an understanding of the regulatory roles of *ACE1* and *DEC1*.

### 5.2 Transcriptional regulation of gibberellin signaling and response of internode growth

Transcription factors play a crucial role in plant growth and development, some of which can regulate internode growth in Poaceae plants through gibberellins. For example, *OsNAC2* is one of the transcription factors in the NAC family. Overexpression of *OsNAC2* in rice has been found to delay flowering time by 5 days in T_3_ generation plants compared to wild-type rice, and exhibits a phenotype of shorter plant height, shorter spike length, and reduced seed setting rate (Chen et al., 2015). The analysis results showed that the expression level of *OsKO2*, a gene involved in gibberellin synthesis, decreased in the transgenic strain, while the expression levels of *OsEATB*, a gene that inhibits gibberellin biosynthesis, and SLRL (SLR1-Like Repressor), a DELLA-like protein that negatively regulates GA signaling by acting as a constitutive repressor. In rice, *OsMADS57*, a MADS-box transcription factor, controls panicle exertion and stem elongation, both of which are essential for vegetative growth (Chu et al., 2019). Because of lower quantities of GA_3_, *OsMADS57* knockdown results in shorter internodes and more sensitivity to GA_3_. By attaching itself to their promoters, it directly inhibits the genes *OsGA2ox3* and *EUI1* (*ELONGATED UPPERMOST INTERNODE 1*) (a gene encoding a cytochrome P450 monooxygenase), which are involved in GA deactivation. Dual luciferase and EMSA assays confirmed its direct regulatory role in GA metabolism. All things considered, *OsMADS57* is a crucial regulator of GA signaling that affects the growth and development of rice. It was discovered that the LBD transcription factor *PheLBD12* controls bamboo height growth. *PheLBD12*’s function in GA signaling was demonstrated by the shorter internodes, decreased GA3 levels, and enhanced GA3 sensitivity observed in rice when it was overexpressed (Wu et al., 2024). *OsGA2ox3* and *OsAP2-39*, which are involved in GA reduction and catabolism, were directly upregulated by *PheLBD12*. Its direct contact with these gene promoters was validated by EMSA, dual-luciferase, and yeast one-hybrid tests. The role of LBD transcription factors in regulating height growth is better understood with this study.

*WRKY* transcription factor family is widely involved in plant growth, development, and stress tolerance (Ahmad et al., 2024). Semi-dwarfism is a key agronomic trait in crop breeding, enhancing lodging resistance and yield stability. A study identified *OsWRKY21*, a group III WRKY transcription factor, as a regulator of rice shoot length through a genome-wide association study (GWAS) (Wei et al., 2021). A quantitative trait nucleotide (QTN) (C/T) was found in the first coding region of *OsWRKY21*, influencing plant height variation in indica rice but not japonica. *OsWRKY21* was widely expressed in embryos, radicles, shoots, leaves, and stems. Overexpression of *OsWRKY21* resulted in semi-dwarf plants with short internodes, early heading, and reduced cell length near the sclerenchyma epidermis, accompanied by decreased indole-3-acetic acid (IAA) and GA3 levels, but increased abscisic acid (ABA) and salicylic acid (SA) levels. The role of *OsWRKY21* was confirmed by the opposite phenotype displayed by CRISPR/Cas9 knockout mutants. Exogenous GA3 treatment at the seedling stage completely restored the semi-dwarf phenotype, demonstrating its function in GA signaling. *OsWRKY21*’s role in GA metabolism (*GA20ox2*, *GA2ox6*, *YABY1*) and cell wall biosynthesis and regulation (*CesA4*, *CesA7*, *CesA9*, *MYB103L*) was demonstrated by RNA-seq and qRT-PCR investigations, connecting it to both growth and stress responses. This study offers important new information about the regulatory function of *OsWRKY21* in internode elongation and plant height, as well as possible precision breeding targets to maximize rice yield and growth.

**Figure 6:**
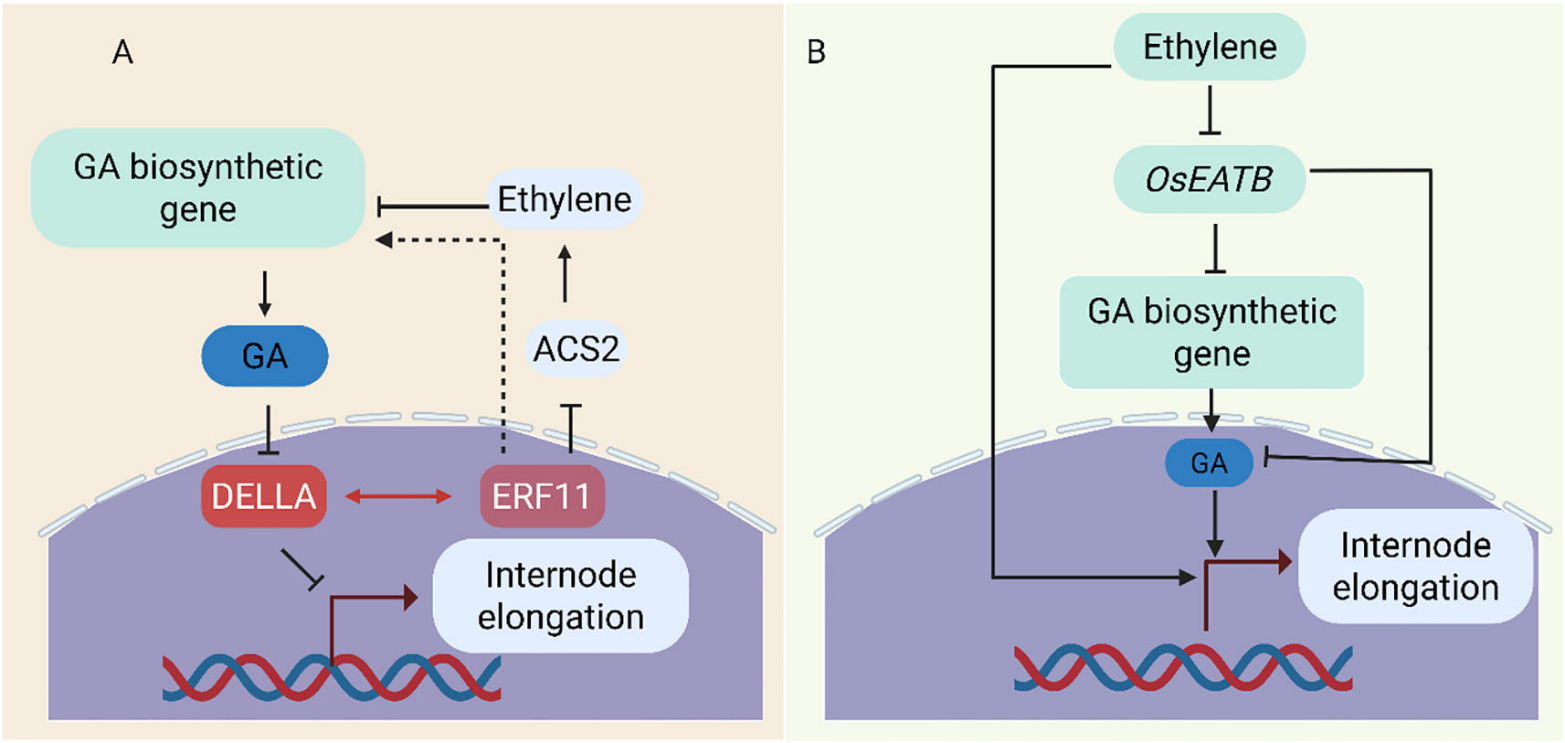
Regulatory mechanisms controlling internode elongation. **(A)** *ERF11* promotes elongation by repressing *ACS2*, lowering ethylene levels, enhancing GA biosynthesis (*GA3ox*/*GA20ox*), and facilitating DELLA degradation. It also directly interacts with DELLA to relieve repression of downstream genes (Zhou et al., 2016). **(B)** By triggering *OsEATB*, which suppresses *OsCPS2* (ent-kaurene synthase A), ethylene prevents elongation, lowering GA biosynthesis and restricting internode growth.

The HD-ZIP transcription factor can affect the expression of genes related to gibberellin biosynthesis. The mutant of rice *small grain and dwarf 2* (*sgd2*) showed a decrease in plant height and seed number. Experiments have found that a deletion of 9 bases in an HD-ZipII family is the cause of the *sgd2* mutant phenotype. The qRT-PCR results showed that the expression levels of genes that positively regulate gibberellin biosynthesis were mostly downregulated in this mutant. This suggests that HD-Zip may affect the level of gibberellin by regulating the expression of genes related to gibberellin biosynthesis, thereby regulating plant growth and development (Chen et al., 2019). ERF11 (ETHYLENE RESPONSE FACTOR) can promote internode elongation in Poaceae plants, and its mechanism of action upregulates the expression of *GA3ox1* and *GA20ox* genes, leading to an increase in the level of bioactive GA (**Figure 6A**) (Zhou et al., 2016). Interestingly, overexpression of *AtERF11* also leads to a decrease in ethylene (CTK) levels, which is consistent with recent findings indicating that *AtERF11* inhibits transcription of the 1-Aminocyclopropane-1-Carboxylate Synthase (ACS) gene involved in CTK biosynthesis. The promoting effect of *AtERF11* on GA biosynthesis gene expression may be achieved through its inhibitory effect on CTK biosynthesis. In another study the *AP2/ERF* transcription factor *OsEATB*, cloned from Indica rice, mediates crosstalk between these hormones (Qi et al., 2011) (**Figure 6B**). *OsEATB* suppresses GA biosynthesis by downregulating ent-Kaurene Synthase A, limiting ethylene-induced GA responses and reducing internode elongation. This results in shorter plants with enhanced tillering and spikelet branching. Such traits are valuable for developing high-yield, dwarf rice varieties. However, the study looks at transcript-level analysis and ectopic expression, offering little information about post-transcriptional or environmental modulation of *OsEATB*. Additional functional validation in various genetic backgrounds or field conditions is still required.

Overall, there is relatively limited research on transcription factors involved in gibberellin regulated internode elongation in Poaceae plants beyond rice, highlighting a significant gap in understanding the genetic and molecular mechanisms governing stem growth in economically important cereals and grasses. Most of the research has concentrated on rice, where it has been demonstrated that important TFs including *OsWRKY21*, *OsMADS57*, and *OsNAC2* control GA biosynthesis, signaling, and response, affecting plant height and environmental adaption. However, less is known about GA-related TFs in other Poaceae species such as wheat, maize, sorghum, and bamboo. The absence of genome-wide functional investigations that identify and describe TFs implicated in GA-mediated internode elongation across various Poaceae species is one significant restriction. Their investigation is further complicated by species-specific regulatory networks and functional redundancy within TF families. Enhancing plant design, improving lodging resistance, and optimizing biomass production, all of which are essential for food security are made possible by further research into these systems. Researchers can find novel TFs controlling GA responses by combining cutting-edge genomic technologies like transcriptome profiling and CRISPR-based gene editing. This could result in crop enhancement tactics that increase major cereal crops’ production, stress resilience, and adaptability. Breeding climate-resilient crops, which would guarantee steady food supply in the face of shifting environmental conditions, may likewise depend heavily on an understanding of GA-TF interactions.

## 6 Epigenetic regulation of internode elongation by gibberellins

**Figure 7:**
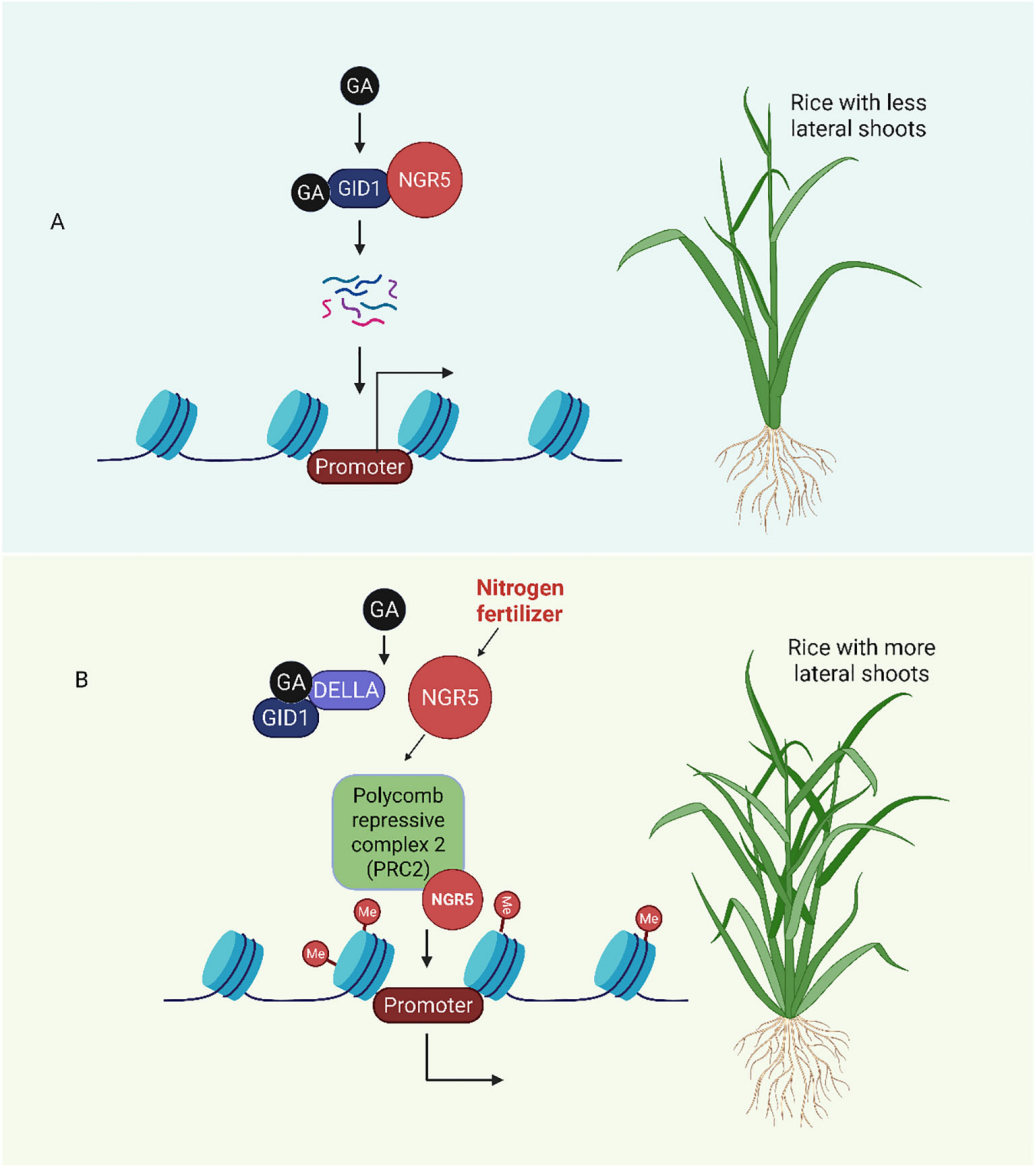
NGR5 integrates nitrogen and gibberellin signals to regulate rice tillering (lateral shoot formation). **(A)** GA binds to GID1, which promotes the degradation of NGR5 and reduces the repression of branching inhibitors. This results in fewer lateral shoots. **(B)** Nitrogen enhances the activity of NGR5, which recruits PRC2 to repress the expression of genes that inhibit branching. Meanwhile, the stabilization of NGR5 against GA by DELLA proteins promotes tillering (Wu et al., 2020).

The precise regulation of gibberellin metabolism and signaling is essential for plant development and environmental responses. Epigenetic regulatory mechanisms, such as histone modification, noncoding RNA-mediated regulation, chromatin remodeling and DNA methylation, are emerging as important means of fine-tuning gene expression (Shan et al., 2025a, 2025b). Recent studies have significantly improved our understanding of the relationships between epigenetic regulation and GA metabolism and signaling (Xie and Chen, 2020). For example; in earlier study, *NITROGEN-MEDIATED TILLER GROWTH RESPONSE 5* (*NGR5)* was identified as a dual-function regulator linking nitrogen metabolism and GA mediated chromatin remodeling in rice. It can interact with the gibberellin receptor GID1 protein. GA uses the ubiquitin-proteasome pathway to encourage the degradation of *NGR5*. Because of this degradation, the PRC2 (polycomb repressive complex 2) complex is less likely to be recruited, which lowers the amounts of H3K27me3 (Histone H3 lysine 27 trimethylation) at particular target gene loci. As a result, GA-mediated developmental responses are facilitated by increased transcriptional activation of growth-related genes. Notably, it has been demonstrated that overexpressing NGR5 preserves the desired semi-dwarf and high-yield characteristics in rice, underscoring the crop’s potential for improvement (**Figure 7**).

According to another investigation, OsYAF9 and OsSWC4 are important subunits of the SWI2/SNF2-Related 1 Chromatin Remodeling (SWR1) and Nucleosome Acetyltransferase of H4 (NuA4) chromatin remodeling complexes that regulate vegetative and reproductive development in rice (Ikram et al., 2022). *OsYAF9* loss resulted in lower plant height, fewer tillers, pollen grain defects, and impaired embryogenesis and seed filling, while *OsSWC4* mutants showed severe pollen germination defects and sterility. Additionally, *OsSWC4* knockdown caused shorter internodes because of impaired cell elongation, a phenotype that was resolved by GA treatment, suggesting that OsSWC4 is involved in the GA biosynthesis pathway. To increase GA gene expression, OsSWC4 mechanistically binds directly to AT-rich areas of GA biosynthetic genes, promoting H2A.Z deposition and H4 acetylation in tandem with OsYAF9. Though the precise chemical connections and functional redundancy with other chromatin modifiers are still unknown, the study offers new insights into chromatin-mediated GA control in rice growth. In order to boost agricultural output and resilience and contribute to food security through targeted genetic and epigenetic alterations in Poaceae species, future research should examine how these complexes integrate environmental cues for optimizing plant design.

Because of the intricacy of epigenetic mechanisms, the dearth of model systems outside of rice, and the technological difficulties involved in examining chromatin dynamics in monocots, there is little research on the epigenetic regulation of internode elongation by GAs in Poaceae species. Histone alterations, chromatin remodeling, and DNA methylation are some of the regulatory layers involved in internode elongation; however, only a few numbers of chromatin remodelers, including SWR1 and NuA4, have been functionally connected to GA biosynthesis. Exact epigenetic mapping is made more difficult by the dynamic and tissue-specific character of GA-induced elongation, and genome-wide studies are hampered by the vast and repetitive genomes of species like wheat and bamboo. Furthermore, sophisticated methods like ChIP-seq and single-cell epigenomics require a lot of resources and are not yet commonly used on grasses. By improving plant architecture in fast-growing species like bamboo, improving lodging resistance in cereals through targeted manipulation of internode elongation, and creating crop improvement strategies based on epigenetics that offer an alternative to genetic modifications, further research in this field holds great promise for food security. Novel regulatory pathways that improve crop resilience and productivity under climate stress may be unlocked by future research combining multi-omics techniques and comparative epigenomic analysis across Poaceae species.

**Figure 8:**
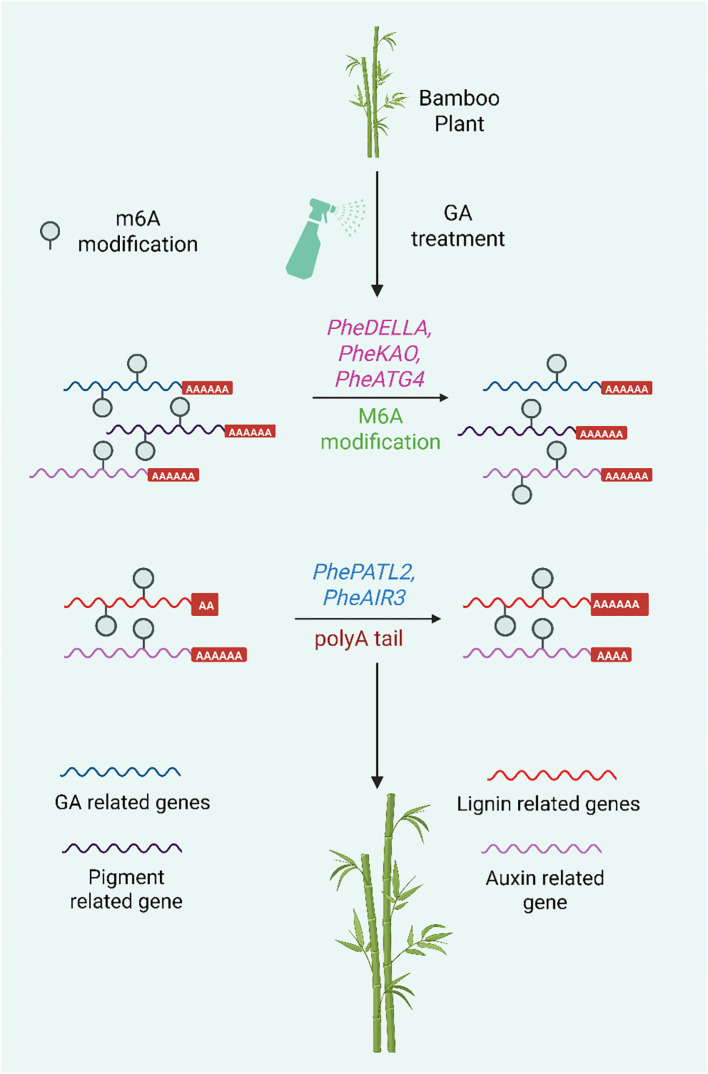
Epigenetic regulation of gene expression by gibberellin (GA) treatment in bamboo during growth, focusing on two key RNA-level modifications: m^6^A modification (N6-methyladenosine) and Poly(A) tail length (PAL).

A recent study revealed that Gas contribute to the rapid growth of Moso bamboo by modulating epigenetic mechanisms (Wang et al., 2025) (**Figure 8**). With an emphasis on RNA modifications such N6-methyladenosine (m6A) and poly(A) tail length (PAL), this work investigates the epigenetic mechanisms by which GA controls the fast growth of Moso bamboo. The researchers established a complex hormonal regulatory network that drives internode elongation by using Nanopore direct RNA sequencing (DRS), which enables real-time detection of RNA modification such as m6A and poly(A) tail length in genes involved in GA biosynthesis, metabolism, auxin transport, and abscisic acid signaling. These results reveal a hitherto undiscovered layer of growth regulation in bamboo and offer crucial insight into how epigenetic changes adjust gene expression in response to GA. Understanding the post-transcriptional control of hormone-responsive genes is crucial for modifying plant architecture and biomass accumulation, which is the justification for our effort. Such information can guide breeding and biotechnology approaches meant to increase crop productivity, and it is particularly useful for creating fast-growing, high-biomass crops or forestry species. Ultimately, by identifying molecular targets that may be used to enhance growth rates and yield potential in agriculturally significant species, this research advances the larger objective of attaining food and resource security.

Another study, explores the role that microRNAs (miRNAs) play in the epigenetic regulation of rapid internode elongation in Moso bamboo (Wang et al., 2021). In comparison to the top section (F1), the lower segment (F4) showed higher amounts of growth hormones, such as auxin cytokinin and gibberellin, as well as increased cell division activity. Analysis of the transcriptome showed that F4 had higher expression of genes involved in cell division and DNA replication. According to degradome analysis and functional validation, 63 differentially expressed miRNAs were found, several of which controlled transcription factors and hormone-responsive genes. Finding post-transcriptional, miRNA-mediated modulation of hormone signaling during spatially controlled internode elongation is the motivation for this work. With new molecular targets for improving biomass accumulation in quickly growing species like bamboo and possibly helping to identify solutions for sustainable resource and food security, these findings reveal a critical epigenetic layer of GA-regulated growth. Future research should focus on applying single-cell epigenomic techniques in Poaceae, integrating transcriptomic and epigenetic datasets to reveal tissue-specific regulatory networks driving internode elongation.

## 7 Crosstalk between GA and other phytohormones

GAs interacts with various phytohormones, including auxins, cytokinins, abscisic acid (ABA), ethylene, jasmonic acid (JA), and Brassinosteroid (BR) to regulate plant growth and development. Besides GA, JA, ABA, BR and auxin signaling pathways are also involved in plant height regulation in Poaceae plants (Cheng et al., 2022). The GA-GID-DELLA pathway is a key regulatory module that controls plant growth and development (Davière and Achard, 2013; Ritonga et al., 2023). DELLA protein serves as a core factor to connect these signaling pathways, coordinating GA signals with other plant hormone signals to jointly regulate plant growth (Ritonga et al., 2023). Numerous studies have shown that auxin can promote the synthesis of active gibberellin and inhibit its degradation, thereby promoting stem elongation (Wolbang et al., 2004). A study in rice has found that the reduction of indole-3-acetic acid in spikelets leads to downregulation of *GA3ox2* transcription levels and loss of *GA1* in the inverted segment, resulting in a decrease in cell number and length, ultimately leading to shortening of the inverted segment and panicle enclosure phenomenon (Yin et al., 2007). By reducing the elongation rate and duration while promoting growth, ethephon treatment decreased the elongation of maize internodes (Zhang et al., 2020). It changed cell size, controlled the expression of the genes for expansin and cellulose synthase, and promoted the formation of lignin through increased activity of phenylalanine ammonia-lyase and peroxidase. Gibberellin and auxin levels decreased because of ethylene signaling’s suppression of *ZmPIN1a*, *ZmPIN4*, and *ZmGA3ox1* and increase of *GA2ox3* and *GA2ox10* transcripts. Transcription factors implicated in cell wall remodeling and ethylene, gibberellin, and auxin signaling were found through transcriptome analysis. These findings suggest that ethylene modulates auxin and gibberellin signaling to regulate cell elongation in maize internodes.

GA promotes stem elongation by triggering DELLA protein degradation. AtERF11, an ERF/AP2 transcription factor, was identified as a positive regulator of GA biosynthesis and signaling in *Arabidopsis* (Zhou et al., 2016). AtERF11 is a member of the ERF subfamily VIII-B-1a of ERF/AP2 transcription factors in *Arabidopsis*. Overexpression of AtERF11 upregulated *GA3ox1* and *GA20ox* genes, increasing bioactive GA levels and GA response, while loss-of-function mutants showed reduced GA response. AtERF11 directly interacts with DELLA proteins to enhance GA signaling and also represses ethylene biosynthesis by downregulating ACS genes. Thus, AtERF11 promotes internode elongation by simultaneously activating GA pathways and inhibiting ethylene production. The ABA content in the uppermost internode of deep rice was significantly decreased and the GA1 content was increased by both water immersion and cytokinin treatment within 3 h. Therefore, it was speculated that the elongation of internodes in deep water rice after immersion is mainly due to the balance between ABA and GA1 mediated by cytokinin (Hoffmann-Benning and Kende, 1992). Qiu et al.’s study also showed that exogenous GA3 stimulated internode elongation in sugarcane by increasing endogenous GAs levels and reducing ABA and ethylene levels (Qiu et al., 2019).

**Figure 9:**
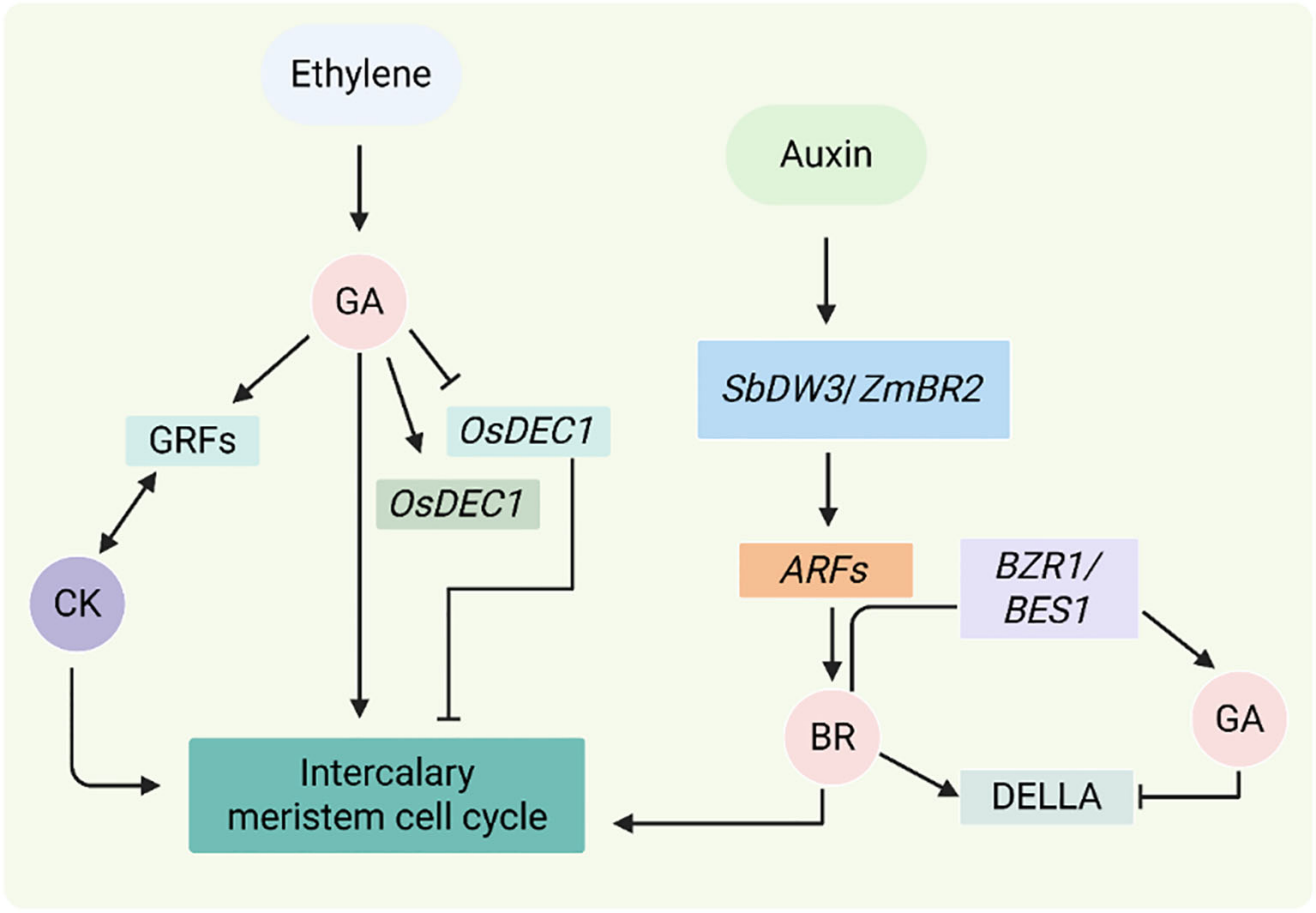
Hormonal regulation of intercalary meristem and internode elongation. The figure shows how GA, IAA, BR, CK, and ethylene regulate the intercalary meristem hormonally. Ethylene increases GA signaling, which controls *OsDEC1* and *OsACE1* to promote cell division, by activating *OsSK1*/*OsSK2*. While BR activates *BZR1*/*BES1* and antagonistically interacts with DELLA, GA works with CK through GRFs to promote growth by degrading DELLA. ARFs are modulated by auxin-BR interactions through *SbDW3*/*ZmBR2* and *SbDW1* to regulate meristem activity. Interactions between antagonistic and synergistic hormones regulate internode elongation, which affects crop biomass and height (Awale and McSteen, 2023).

BR is an important phytosteroid with a wide range of biological activities that play important roles in many aspects of plant development, such as promoting growth, cell division and elongation, and enhancing plant stress resistance (Chakraborty et al., 2025). GA and BR can coordinate to control cell elongation and plant height in rice (Sun et al., 2015) (**Figure 9**). On the one hand, BRs promote GA synthesis by regulating transcription factors. Specifically, BRs inhibit the activity of *GSK3*-like kinases (negative regulators), which allows the BZR1 transcription factor to enter the nucleus. Once in the nucleus, BZR1 partially activates its downstream targets, thereby stimulating the expression of genes involved in GA biosynthesis. The increase in GA level leads to the degradation of another negative regulator DELLA protein, thereby further activating *BZR1* activity to regulate gene expression and promote plant growth (Tong et al., 2014). On the other hand, GA signaling assists in the release of *BZR1/BES1* (*Brassinosteroid Insensitive 1 EMS Suppressor 1*) to promote plant growth. In other words, DELLA, GA, and GA receptor GID1 form a complex, and then under the action of GID2 and 26S proteasome, DELLA protein is degraded, and the inhibition of *BZR1* or *BES1* by DELLA protein is released, ultimately promoting cell elongation (Bai et al., 2012; Li et al., 2012). Initially, this was thought to be the point of intersection between the GA and BR signaling pathways. According to recent research, GA is influenced by BRs both metabolically and at the signaling level in *Arabidopsis* and rice. By upregulating GA20ox-2 and GA3ox-2 and suppressing GA2ox-3, BR enhances the production of bioactive GA1 and cell elongation in rice, hence promoting GA biosynthesis. BZR1 and several BR-related transcription factors, such as DLT and *RLA1/SMOS1*, influence this control. On the other hand, excessive BR (from external brassinolide treatment, for example) can suppress BR production via TFs such *OFP1* and activate GA-inactivating genes, which might hinder growth. Through their targeting of GA oxidase genes, OFP proteins adversely control GA production in both *Arabidopsis* and rice (Tong et al., 2014).

**Figure 10:**
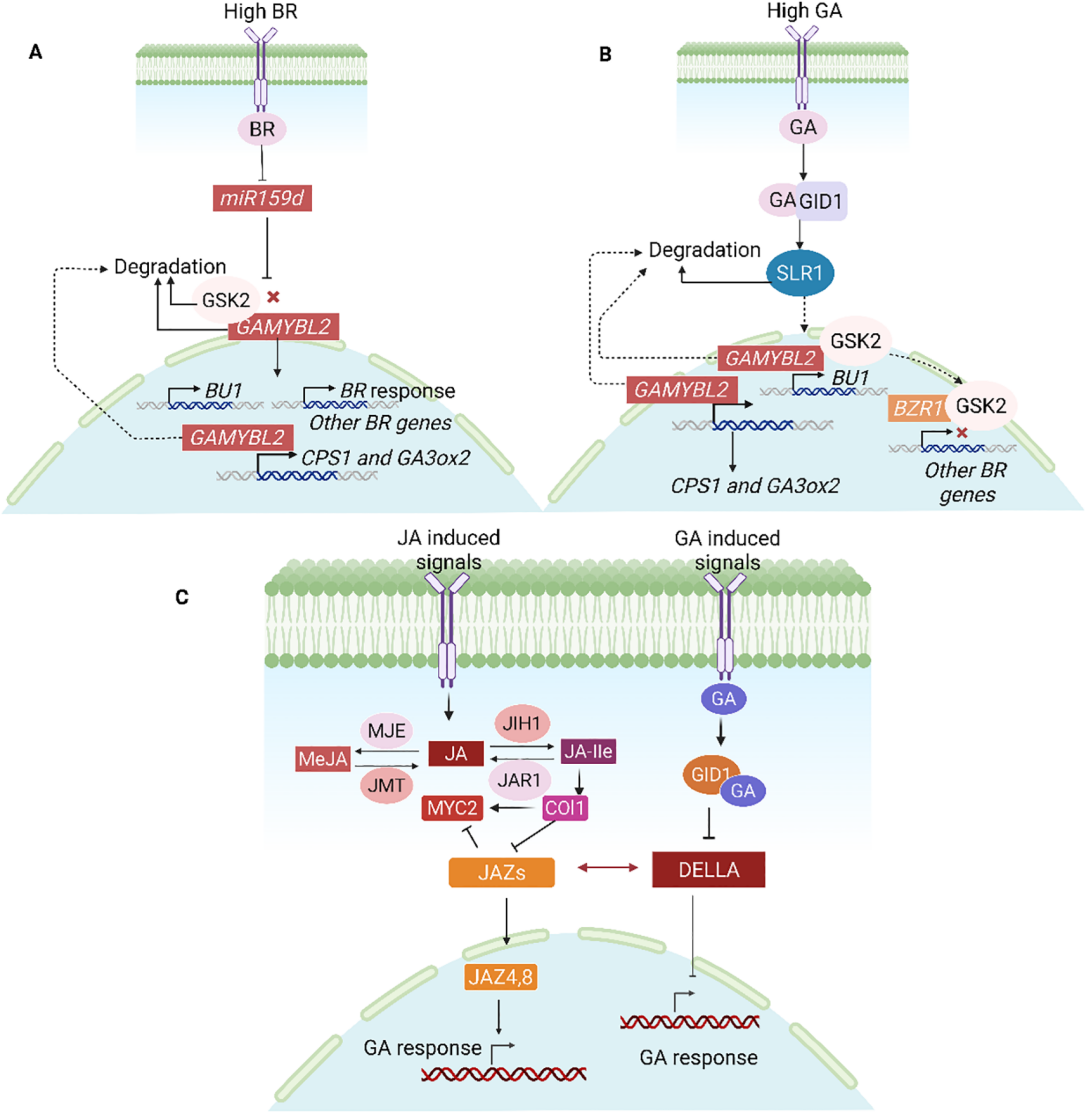
Hormonal interplay modulating gene expression and signal transduction. **(A)** High BR decreases *miR159d*, which leads to the degradation of *GAMYBL2* and *GSK2*, which in turn triggers the activation of BR-responsive genes, GA biosynthesis, *BU1*, *CPS1*, and *GA3ox2*. **(B)** While *GSK2* inhibits BR responses, high GA degrades *SLR1* and *GAMYBL2*, triggering GA signaling and target genes. **(C)** Through DELLA-JAZ dynamics, GA and JA interact: stable DELLAs allow the activation of the JA pathway at low GA, while GA-mediated DELLA degradation enables JAZs to repress *MYC2*, suppressing JA signaling. This regulatory mechanism highlights the complex crosstalk between JA and GA in plant growth and stress responses (Gupta et al., 2023).

In a recent work it was found that BR inhibits OsmiR159d, which causes its target *OsGAMYBL2* to accumulate (Gao et al., 2018) (**Figures 10A, B**). This *OsGAMYBL2* in turn controls the expression of BU1, which is involved in BR signaling, as well as ent-COPALYL DIPHOSPHATE SYNTHASE 1 (*CPS1*) and *GA3ox2*, which are involved in GA biosynthesis. Crucially, SLR1, a GA signaling repressor, decreases the transcriptional activity of *OsGAMYBL2*, whilst *OsGSK2* stabilizes it under BR treatment. On the other hand, GA signaling indirectly increases BR signaling by encouraging the breakdown of *OsGAMYBL2*. This demonstrates how important *OsGAMYBL2* is in bridging the gap between GA biosynthesis and BR signaling. The goal of this work is to better understand how plants fine-tune hormone networks to control growth responses by addressing the previously unknown crosstalk between rice’s BR and GA pathways.

The signaling pathways for GA and JA work together to regulate developmental processes and stress reactions (**Figures 10C**). The repressors of each pathway, the DELLA and JASMONATE ZIM-DOMAIN PROTEIN (JAZ) proteins, interact to orchestrate this coordination, which impacts plant growth and yield. GA and JA antagonize and regulate the growth and development of plants, as well as their response to biotic and abiotic stresses. JAZ (Jasmonate ZlM Domain) is a key inhibitor in jasmonic acid signaling, which triggers ubiquitination and degradation of JAZ factors. In *O. sativa* (rice), direct physical interaction between *JAZ8/JAZ9* and the DELLA protein SLR1 have been confirmed (Um et al., 2018). When the JA signal is downregulated, JAZ accumulates and DELLA protein activity is inhibited, thereby relieving the inhibition of the GA signaling pathway by SLR and enhancing the expression of GA signal responsive genes, ultimately promoting plant growth (Um et al., 2018). The DELLA–JAZ crosstalk is an attractive target for crop development because it offers a useful regulatory node for balancing growth and stress responses. Stress tolerance may be increased by modifying elements like SLR1 (DELLA) or JAZ repressors (e.g., *OsJAZ9*) without significantly lowering plant height. To maximize the growth-defense trade-off in rice and other cereals, for instance, partial reduction of DELLA activity or fine-tuning JAZ expression have been investigated. For breeding programs looking to increase yield stability under stress, these interactions provide specific molecular targets.

In summary, the complex interactions among the BR, GA, and JA signaling pathways are essential for controlling the growth, development, and stress reactions of plants. Through transcriptional regulation, BR stimulates GA production and signaling, whereas GA increases BR signaling by removing DELLA-mediated repression. By regulating gene expression for growth regulation, *OsGAMYBL2* becomes a key integrator between BR signaling and GA metabolism. Furthermore, the complex hormonal coordination affecting plant height and stress tolerance is further highlighted by the interaction between the DELLA and JAZ proteins. The purpose of this work is to clarify the intricate hormonal network in poaceae, such as rice, providing targets for agronomic and genetic approaches to enhance crop performance.

## 8 Conclusions and future prospects

This review underscores the central role of GA in regulating internode elongation across Poaceae species, with a particular emphasis on the unique molecular architecture of bamboo. We uncover how GA controls elongation by activating downstream genes involved in cell division, cell wall remodeling, and elongation by investigating the hormone’s biosynthesis, metabolic regulation, and signaling pathways. Although bamboo belongs to the grass family, it differs from traditional cereal crops due to its unique anatomical and developmental traits, including rapid culm elongation bursts, seasonally synchronized growth, and synergistic hormonal crosstalk, especially between GA, auxin, and brassinosteroids.

Deciphering the fundamental GA biosynthesis and signaling pathways has advanced significantly, yet there are still many unanswered questions. Since these components are essential for regulating genes involved in cell elongation and division, future studies should focus on clarifying the entire range of GA-responsive transcription factors, protein kinases, and epigenetic regulators. Furthermore, since light, temperature, and humidity are dynamic environmental cues that influence hormone sensitivity and subsequent reactions, GA-mediated development should no longer be investigated in a vacuum (Robil et al., 2024). Moreover, cross-species comparisons leveraging pan-genomic resources can help identify novel alleles that confer desirable internode traits, GA sensitivity, and environmental adaptability (Wang et al., 2025). However, only a limited range of species and genetic variation are covered by the bamboo genomic data available today. Therefore, to enable meaningful allele mining and genotype–phenotype correlations, expanded genome sequencing across diverse bamboo species and ecotype, coupled with high-resolution phenotyping datasets. Additionally, most studies focus on early growth stages, whereas long-term assessments tracking GA’s impact on plant morphology, yield components, and stress resilience throughout the entire life cycle are crucial for practical applications. Furthermore, the potential trade-offs of GA application, such as unintended effects on disease susceptibility, resource allocation, and lodging risk, require careful evaluation.

We support a systems biology framework that combines environmental modelling, AI-driven data analytics, and multi-omics (such as transcriptomics, proteomics, and metabolomics) to address the complexity of internode elongation in field settings (Wang et al., 2021; Zhang et al., 2025). For example, network biology can uncover GA-regulated hubs driving phenotypic variation, and machine learning models can be trained on multi-layered omics and environmental data to anticipate elongation dynamics or identify important regulatory modules. To operationalize these methods into practical breeding and culture tactics, we urge interdisciplinary cooperation, as such integrative efforts in bamboo are still poorly unexplored.

In conclusion, elite bamboo varieties with optimized culm architecture, increased mechanical strength, and improved environmental resilience will be possible once the GA-mediated internode elongation networks in bamboo are deciphered within the larger framework of Poaceae evolution and physiology. In a changing climate, precise breeding and sustainable farming of bamboo and associated grass species will require a systems biology framework that combines environmental modelling, multi-omics, and AI-driven analytics.
